# A disulfidptosis-related lncRNA signature for predicting prognosis and evaluating the tumor immune microenvironment of lung adenocarcinoma

**DOI:** 10.1038/s41598-024-55201-7

**Published:** 2024-02-26

**Authors:** Zipei Song, Xincen Cao, Xiaokun Wang, Yuting Li, Weiran Zhang, Yuheng Wang, Liang Chen

**Affiliations:** 1 Department of Thoracic Surgery, The First Affiliated Hospital of Nanjing Medical University, Nanjing, China; 2 The First Affiliated Hospital of Nanjing Medical University, Nanjing, China; 3 Department of Graduate Administration, Chinese PLA General Hospital, Beijing, China

**Keywords:** Lung adenocarcinoma, Disulfidptosis, LncRNA, Prognosis, Tumor immune microenvironment, Cancer, Lung cancer, Non-small-cell lung cancer, Computational biology and bioinformatics, Databases, Genome informatics, Machine learning

## Abstract

As a novel form of regulated cell death (RCD), disulfidptosis offering a significant opportunity in better understanding of tumor pathogenesis and therapeutic strategies. Long non-coding RNAs (lncRNAs) regulate the biology functions of tumor cells by engaging with a range of targets. However, the prognostic value of disulfidptosis-related lncRNAs (DRlncRNAs) in lung adenocarcinoma (LUAD) remains unclear. Therefore, our study aimed at establishing a prognostic model for LUAD patients based on DRlncRNAs. RNA-seq data and clinical information were obtained from The Cancer Genome Atlas (TCGA) database. Subsequently, a prognostic model based on DRlncRNAs was constructed using LASSO and COX regression analysis. Patients were stratified into high- and low-risk groups based on their risk scores. Differences between the high-risk and low-risk groups were investigated in terms of overall survival (OS), functional enrichment, tumor immune microenvironment (TIME), somatic mutations, and drug sensitivity. Finally, the role of lncRNA GSEC in LUAD was validated through in vitro experiments. Using the prognostic model consists of 5 DRlncRNAs (AL365181.2, GSEC, AC093673.1, AC012615.1, AL606834.1), the low-risk group exhibited a markedly superior survival in comparison to the high-risk group. The significant differences were observed among patients from different risk groups in OS, immune cell infiltration, immune checkpoint expression, immunotherapy response, and mutation landscape. Experimental results from cellular studies demonstrate the knockdown of lncRNA GSEC leading to a significant reduction in the proliferation and migration abilities of LUAD cells. Our prognostic model, constructed using 5 DRlncRNAs, exhibited the capacity to independently predict the survival of LUAD patients, providing the potentially significant assistance in prognosis prediction, and treatment effects optimization. Moreover, our study established a foundation for further research on disulfidptosis in LUAD and proposed new perspectives for the treatment of LUAD.

## Introduction

Lung cancer, a globally malignant neoplasm, stands as the leading contributor to cancer-related mortality^1^. Conventionally, it is classified into two histopathological subtypes: non-small cell lung cancer (NSCLC) and small cell lung cancer (SCLC), with NSCLC accounting for approximately 85% of the total incidence^2,3^. Among NSCLC cases, the most common pathological subtype is lung adenocarcinoma (LUAD). The choice of treatment for LUAD primarily depends on its TNM stage, with surgical resection being the preferred approach for early-stage patients. However, patients with advanced LUAD have a poor prognosis, even with targeted therapy^4^. Therefore, the development of reliable biomarkers and prognostic indicators for early LUAD detection is considered essential to enhance patient outcomes.

Regulated cell death (RCD) plays a pivotal role in both tumorigenesis and the progression of tumors. Furthermore, research focused on Regulatory cell death (RCD) contributed significantly to our understanding of tumor pathogenesis and provided essential strategies for tumor treatments^5,6^. It has been reported that the biological behavior and therapeutic response of LUAD were affected by kinds of RCD, such as apoptosis, necroptosis, pyroptosis, ferroptosis and cuproptosis and so on^7^. Disulfidptosis, recently identified as a novel subtype of RCD, stands apart from other categories of RCD, such as ferroptosis and cuproptosis. Liu et al. observed cancer cells with high SLC7A11 expression, induced by glucose starvation, resisted classification within established cell death categories. This novel form of cell death was unresponsive to common cell death inhibitory drugs and resisted the suppression of key genes associated with ferroptosis or apoptosis. Nevertheless, the application of thiol oxidizers, such as diamide and diethyl maleate, significantly accelerated this unique cell death process, leading to its identification as disulfidptosis^8^. Additionally, the tumor microenvironment (TME) assumes an essential part in the tumorigenesis, immune evasion, and the responses of LUAD treatment^9^. Disulfidptosis has also shown potential effects on immune infiltration^10^, however, the specific role of disulfidptosis in LUAD remains to be further clarified. LncRNAs are defined as transcripts exceeding 200 nucleotides in length that do not code for proteins^11^. Increasing evidence highlights the significant role of lncRNAs in LUAD progression and resistance to therapies through their regulation of RCD^12,13^. However, the mechanisms through which lncRNAs may modulate disulfidptosis in the context of LUAD await further elucidation.

In this research, we conducted a comprehensive exploration aimed at assessing the clinical significance of disulfidoptosis and lncRNAs in LUAD. Firstly, we established a disulfidoptosis-related lncRNA-based signature, denoted as DRLS, by employing LASSO analysis and Cox analysis. Secondly, a series of analyses were conducted to investigate functional enrichment, immune cell infiltration, immunotherapeutic potential, drug sensitivity, and somatic genomic mutations among patients characterized by different DRLS scores. Subsequently, cellular experiments were conducted to validate the differential expression of five lncRNAs which were utilized in the construction of DRLS. Finally, the results of in vitro experiments confirmed the inhibitory impacts on LUAD cell proliferation and migration upon GSEC knockdown. In summary, our study introduced a novel prognostic indicator for LUAD patients and provided valuable guidance for therapeutic decision-making, meanwhile shed light on the potential mechanisms involving DRlncRNAs in LUAD.

## Materials and methods

### Data acquirement and processing

The transcriptomic data, somatic mutation data, and clinical information of LUAD were obtained from the Cancer Genome Atlas (TCGA) database (http://portal.gdc.cancer.gov/) and then under pretreatment for subsequent analyzes. The copy number variation (CNV) information was downloaded from Xena database (http://xena.ucsc.edu/)^14^. Ultimately, a cohort consisted of 507 patients was included in our investigation and was randomly divided into the training set (355 patients) and testing set (152 patients) at a ratio of 7:3 using the R package “caret”. The comparative analysis of clinical information between these two sets was performed utilizing the chi-square test methodology.

### Identification of disulfidptosis-related lncRNAs

According to present research^8^, 16 genes which implicated in disulfidptosis pathways were selected and denoted as disulfidptosis-related genes (DRGs) (Table S1). Utilizing a lncRNA annotation data from the GENCODE database (http://www.gencodegenes.org/), a comprehensive cohort of 16,876 lncRNAs linked to disulfidptosis processes was extracted by the use of the Perl programming language. Subsequently, a comprehensive set of 104 lncRNAs associated with disulfidoptosis was identified and validated through Pearson’s correlation analysis (|Pearson R| > 0.4, P < 0.001).

### Building and evaluating the DRLS

The training set served as the foundational basis for constructing the DRLS, while the testing and entire sets were employed to assess and validate the prediction accuracy of the DRLS. Prognostic lncRNAs were screened and examined through a series of analytical methodologies, encompassing univariate and multivariate Cox regression analysis^15^, LASSO regression analysis^16^, by utilizing the “glmnet” package. Additionally, the DRLS score for each LUAD patient was computed via the formula as follow:

$${\text{DRLS score }} = \, \sum {\text{Coef lncRNAs }} \times {\text{ Expr lncRNAs}}.$$

Coef and Expr represent the coefficient and the DRlncRNAs expression respectively.

Patients in the training, testing as well as entire sets were respectively categorized to the low and high risk groups based on the DRLS score by using the “survminer” package. Subsequently, the Kaplan–Meier (K–M) analysis and log-rank test were employed for the assessment of disparities in overall survival (OS) among these two groups, along with using the “survival” package for statistical analyses. Receiver operating characteristic (ROC) was applied for the accuracy evaluation of the developed signature in predicting OS of LUAD patients using the package “timeROC”. Principal Component Analysis (PCA)^17^ and t-distributed Stochastic Neighbor Embedding (t-SNE) were applied for further facilitating dimensionality reduction and visualize the distinctions between these two risk groups. Furthermore, univariate and multivariate Cox regression analyses were employed to evaluate the independent prognostic significance of the DRLS score.

### Construction of the nomogram

Nomograms were constructed through the combination of the risk scores and clinical variables including gender, age, and stage, with the aim of predicting the 1-, 3-, and 5-year OS of LUAD patients. The nomograms were developed using the “RMS” package. Furthermore, the Hosmer–Lemeshow test was applied to generate calibration plots for depicting the assistance between actual outcomes and predictive results. Additionally, we generated Decision Curve Analysis (DCA) curves and ROC curves for further validation of the nomogram.

### Functional enrichment analysis

The “LIMMA” R package was employed to discern the differentially expressed genes (DEGs) in the high and low DRLS groups (|Log2FC| > 2.0, P < 0.05). Subsequently, functional analyses encompassing Gene Ontology (GO) and Kyoto Encyclopedia of Genes and Genomes (KEGG)^18–20^ were carried out utilizing the ‘ClusterProfiler’, ‘org.Hs.eg.db’, ‘enrichplot’, ‘ggplot2’, ‘RColorBrewer’, ‘dplyr’, ‘ggpubr’, and ‘ComplexHeatmap’ packages. The “GSVA” R package was used to conduct Gene Set Variation Analysis (GSVA) for exploring potential variations in biological functions. The gene set files utilized for these analyses were acquired from the Molecular Signatures Database or previously published research. Additionally, the “c2.cp.kegg.Hs.symbols.gmt” gene set file was employed as a reference dataset for performing Gene Set Enrichment Analysis (GSEA).

### Analysis of tumor mutation burden (TMB)

The “maftools” package was employed to quantify tumor mutation burden (TMB). Following this, all LUAD patients were stratified into two groups based on distinct TMB levels determined by the median TMB score. Additionally, the association between DRLS and TMB was investigated using Spearman correlation analysis.

### Evaluation of the tumor immune microenvironment

Firstly, the “ESTIMATE” R package was applied for calculation of the immune scores, stromal scores, and ESTIMATE scores of each patient to evaluate the population of immune cells and stromal cells between distinct groups. And then, the relationship between the three scores and DRLS were further evaluated using Spearman correlation analysis. Subsequently, 8 distinct algorithms, including TIMER, xCELL, CIBERSORT, CIBERSORT-ABS, quanTIseq, MCP-counter, EPIC, and ssGSEA were employed to access the infiltration of immune cells^21–28^. Finally, the comparative analysis focused on the immune checkpoints (ICs) expression was conducted to explore the potential utility of DRLS in predicting responses to immunotherapy.

### Immunotherapeutic response prediction and drug sensitivity

The tumor immune dysfunction and exclusion (TIDE) algorithm was accessed and utilized online (http://tide.dfci.harvard.edu/)^29^. The lower TIDE scores tend to associated with more favorable responses toward Immune Checkpoint Inhibitors (ICIs) therapy. Immunophenoscores (IPS) for LUAD were acquired from the Cancer Immunome Atlas (TCIA) database (http://tcia.at/home)^30^. The Cancer Drug Sensitivity Genomics of Cancer Cell Lines (GDSC) database (https://www.ancerrxgene.org/) were applied for assessment of potential clinical application of DRLS, along with the calculation of half-maximal inhibitory concentration (IC50) for commonly used anti-tumor drugs using the R package “pRRophetic”. The predictive results were estimated applying ridge regression analysis, as for the prediction precision was measured via tenfold cross-validation.

### Consensus clustering analysis

All patients diagnosed with LUAD were stratified into two clusters based on the expression levels of DRlncRNAs, employing the “ConsensusClusterPlus” R package. This classification aimed to investigate potential molecular subtypes. Following this, distinctions between the two clusters regarding survival rates, time-related parameters, immune infiltration, and response to immune therapy were assessed using the methodologies previously outlined.

### Cell lines culture

The human bronchial epithelial cell line BEAS-2B and two human lung adenocarcinoma cell lines (A549 and H1975) were procured from the Institute of Biochemistry and Cell Biology of the Chinese Academy of Sciences. All the cells were cultured in RPMI-1640 medium (Gibco; Thermo Fisher Scientific, Inc.) supplemented with 10% fetal bovine serum (FBS) (Gibco; Thermo Fisher Scientific, Inc.) and 1% penicillin–streptomycin. The culture conditions were maintained at a temperature of 37 °C, a CO_2_ concentration of 5%, and a humidity level of 95%.

### Cell transfection

Short interfering RNAs (siRNAs) were synthesized by KeyGEN (Nanjing, China). Transfection procedures were performed employing Lipofectamine 2000 (Invitrogen, USA), following the manufacturer’s instructions. The assessment of transfection efficiency was conducted 48 h after transfection, utilizing quantitative real-time polymerase chain reaction (qRT-PCR). The target sequences are as follows:

- GSEC si-1:5′-AGCTACCAGATTCCTTGTGAA-3′;
- GSEC si-2:5′-GACTGGCTGATATCCAACTAT-3′.

### RNA extraction and quantitative real-time polymerase chain reaction assays

Total RNA was extracted from the cells using TRIzol® reagent (Invitrogen; Thermo Fisher Scientific, Inc.) in accordance with the manufacturer’s instructions. Subsequently, reverse transcription was carried out utilizing a PrimeScript Reverse Transcription Kit (Takara Bio, Inc.). For RT-qPCR, an StepOnePlus™ Real-Time PCR System (Applied Biosystems; Thermo Fisher Scientific, Inc.) was employed. The conditions for the RT-qPCR reaction involved an initial step at 95 °C for 5 min, following 40 cycles comprising denaturation at 95 °C for 15 s, annealing at 60 °C for 30 s, and extension at 72 °C for 45 s. The 2^−ΔΔ^Ct computing method was employed to calculate the relative mRNA expression, which was normalized using β-actin as a reference. All reactions were conducted in triplicate. The gene primers applied within the study are as follows:

AL365181.2: F:5′-ACACTTGGCCATATGTGTCTTC-3′;
  R:5′-TGAGACAGGAGGTTCTACCTGA-3′;
GSEC: F:5′-CTGAGCTACCAGATTCCTTGTG-3′;
  R:5′-GCTCATCTGCAGAAGGTCTAAG-3′;
AC093673.1: F:5′-CAAATCCGGCTACACTCTACTG-3′;
  R:5′-CAACCAGATCTGACACCAGTTT-3′;
AC012615.1: F:5′-TGTGTTCAGGTGCACAGAGTAG-3′;
  R:5′-GCCCCAAGTAAAGTAACTGACC-3′;
AL606834.1: F:5′-ACAAAGGAGAGGGAAGAGTCAG-3′;
  R:5′-ATATAGGTGGGAAAGGTGAACG-3′.

### Cell counting kit-8 (CCK-8) experiment

The CCK-8 assay was conducted employing CCK-8 solution (Dojindo Laboratories, Inc.), in strict accordance with the manufacturer’s instructions. Cells were seeded into 96-well plates at a density of 2 × 10^3^ cells/100 µl per well. Following incubation periods of 0, 24, 48, 72, or 96 h, 10 µl CCK-8 per well solution was added to the 96-well plates to measure the relative number of viable cells, with the absorbance at 450 nm was evaluated by a microplate reader.

### Colony formation

In the colony formation assay, LUAD cells were seeded in 6-well plates at a density of 1 × 10^3^ cells/well. Subsequently, these plates were incubated at 37 °C with a 5% CO_2_ for a duration of 14 days. Following the incubation period, colonies were fixed using 4% paraformaldehyde and stained with 0.1% crystal violet obtained from Beyotime Institute of Biotechnology. The population of colonies in each well was counted, with each colony comprising more than 50 cells. The experiments were performed in triplicate.

### Wound-healing assay

LUAD cells were seeded in 6-well plates when the concentration reached 90%. A linear scratch wound was generated within the monolayers of cells using a 20 µl pipette tip. Subsequently, cells were cultured within an FBS-free medium and followed by carefully washing with PBS to remove debris and suspended cells. The images of each wound were recorded at 0 and 36 h by the use of an inverted microscope.

### Transwell assay

Transwell chambers (8-µm pore size; Corning, Inc.) were applied for the migratory capabilities evaluation of LUAD cells. In each upper Transwell chamber, 4 × 10^4^ cells were seeded with 300 µl of serum-free medium, while the lower chamber was filled with 700 µl of RPMI-1640 medium supplemented with 10% FBS. After a 2-day incubation period, cells were fixed using 4% paraformaldehyde and subsequently stained with 0.1% crystal violet obtained from Beyotime Institute of Biotechnology. Non-migratory cells residing on the upper insert chamber membrane were carefully eliminated using a cotton swab. Following this, images of the stained cells were acquired using an inverted microscope. Cell counts were then performed in five randomly selected fields for the evaluation of the LUAD cells migratory potential. All experiments were replicated in triplicate.

### Statistical analysis

Statistical analyses were conducted using the R, Perl, and GraphPad Prism 8 platforms. Group differences were assessed using Student’s t-test and the Wilcoxon rank sum test. The association between two variables was evaluated using Pearson’s and Spearman’s correlation methodologies. A significance threshold of P < 0.05 was considered indicative of statistical significance in this study.

## Results

### The landscape of DRGs and DRlncRNAs in LUAD

**Figure 1:**
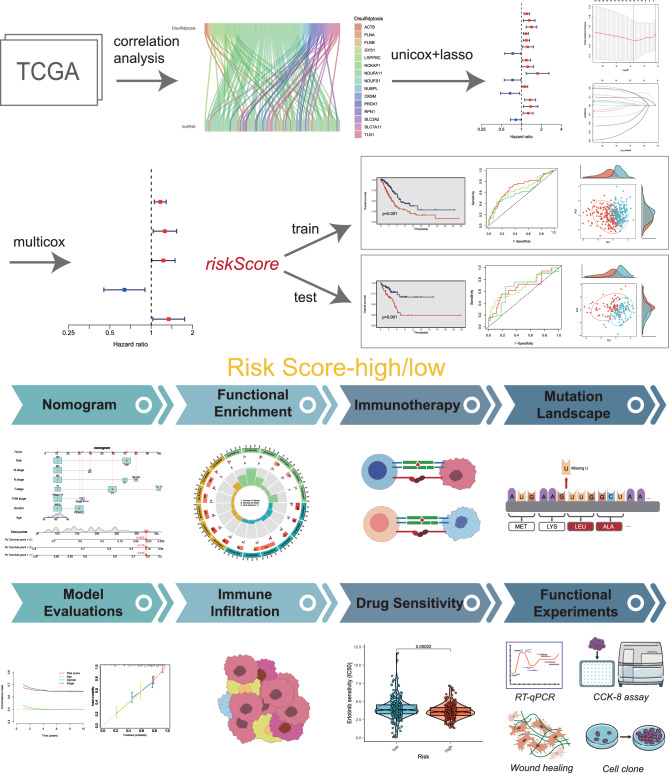
The workflow of this study.

According to the research focused on disulfidptosis, there are 16 key genes which take an important part in the process of disulfidptosis, therefore we selected these 16 disulfidptosis-related genes (DRGs) for further study, Fig. 1 depicted the flowchart of this research.

**Figure 2:**
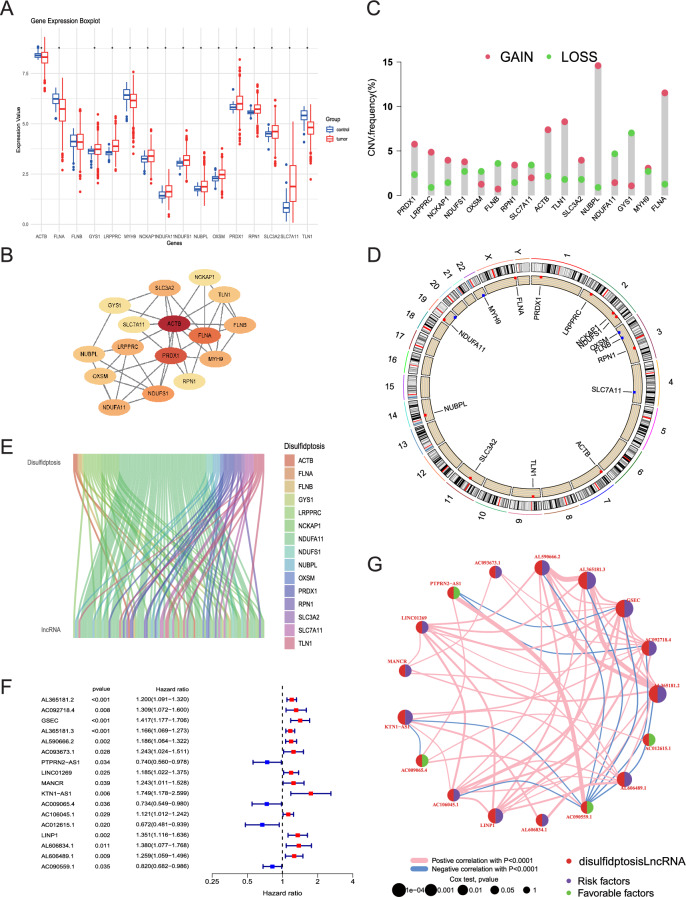
Characteristics of DRGs in LUAD and Identification of DRlncRNAs. (**A**) The differential expression of 16 DRGs between normal and LUAD samples. (**B**) A PPI network established based on 16 DRGs. (**C**) Assessment for the frequency of CNV among the 16 DRGs. (**D**) The circular plot depicts the chromosomal distribution of the 16 DRGs. (**E**) A Sankey diagram presented the co-expression of the 16 DRGs and corresponding lncRNAs. (**F**) The forest plot illustrates the prognostic DRlncRNAs selected through univariate Cox regression analysis. (**G**) The interaction network of the 17 lncRNAs depicted with distinct colors indicating differences in correlations.

Firstly, we studied the expression levels of 16 DRGs in 58 normal samples and 524 LUAD tumor samples from TCGA database (Fig. 2A). It was observed that, except for FLNB, the remaining 15 DRGs displayed differential expression between tumor and normal tissues. Subsequently, protein–protein interaction (PPI) networks for the 16 DRGs were constructed applying the String website and Cytoscape software. And the findings revealed tight interactions among these 16 DRGs (Fig. 2B). In addition, we also investigated the frequency of copy number variation (CNV) mutations, and found that all of the 16 DRGs contained a large number of CNV mutations. The decreased extensive CNVs were observed in OXSM, FLNB, SLC7A11, NDUFA11, GYS1, while the overall enhancement of CNVs were presented in ACTB, TLN1, NUBPL, FLNA (Fig. 2C). We illustrated the CNV alteration locations on the chromosomes for these 16 DRGs (Fig. 2D). Next, we extracted 16,876 lncRNAs from the GENCODE database using Perl-based methods. Subsequently, 104 lncRNAs associated with disulfidptosis were identified by the use of Pearson’s correlation analysis (|Pearson R| > 0.4, P < 0.001, Table S2), and their co-expression network with DRGs was visualized in a Sankey’s plot (Fig. 2E). Furthermore, LUAD patients were involved in our study, which categorized into the training set and testing set. The training set was applied for identifying prognostically relevant LncRNAs and constructing DRLS, while the testing set served as the validation of the predictive efficacy of DRLS. There was no significantly statistic difference observed in the comparative analysis in age, stage, gender, and TNM between the two groups (P > 0.05, Table S3). Subsequently, 17 DRlncRNAs highly correlated with prognosis through univariate COX regression analysis were identified (Fig. 2F). Finally, we generated a prognostic correlation network diagram for these 17 DRlncRNAs (Fig. 2G), identifying all DRlncRNAs as risk factors, except for PTPRN2-AS1, AC009065.4, AC012615.1, and AC090559.1, which were considered protective factors.

### Construction and validation of a prognosis signature in the training set

**Figure 3:**
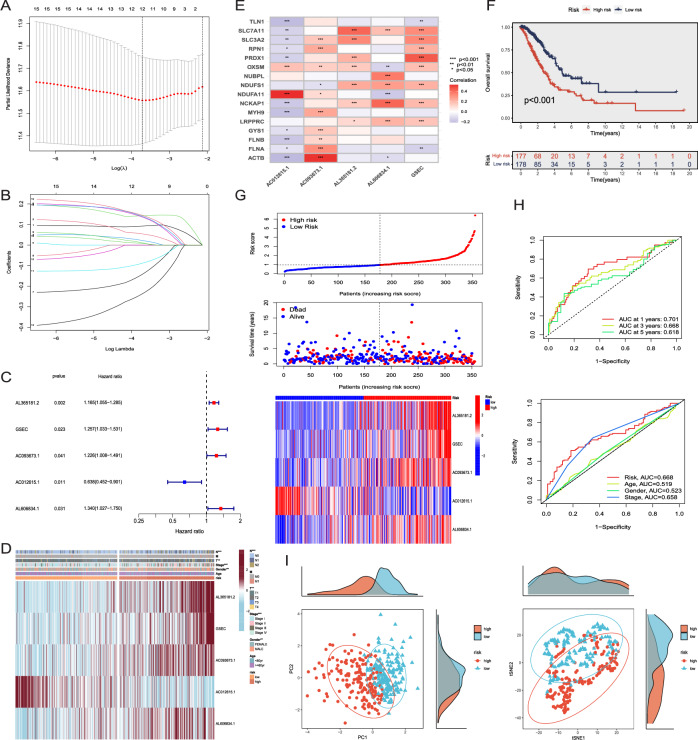
Construction and validation of a prognosis signature in the training set. (**A,B**) Further selection of prognostic DRlncRNAs was performed through LASSO regression analysis. (**C**) 5 DRlncRNAs were screened through the multivariate COX regression analysis. (**D**) A clinical correlation heatmap depicted the association between the prognostic signature of DRlncRNAs and clinical outcomes. (**E**) The correlation analysis for 16 DRGs and 5 DRlncRNAs used to prognostic signature construction. (**F**) K–M analysis for predictive capacity of DRLS. (**G**) The distribution of DRLS scores as well as survival status and time of each LUAD patient, along with the clustering heatmap of the 5 DRlncRNAs. (**H**) ROC curves for predicting the OS of 1, 3, and 5-years, along with clinical ROC curves. (**I**) PCA and t-SNE analyses were performed on LUAD patients based on the risk score.

According to the univariate COX regression analysis, 17 DRlncRNAs were identified and then applied in LASSO regression analysis. Subsequently, 12 DRlncRNAs were identified as significantly linked to the LUAD patients prognosis using the optimal lambda value (Fig. 3A,B). Finally, 5 DRlncRNAs were determined via multivariate COX regression analysis (Table S4). Among these, AC012615.1 was a prognostic protective factor, while AL365181.2, GSEC, AC093673.1, AL606834.1 were prognostic risk factors (Fig. 3C). In addition, we examined the correlation of the five DRlncRNAs with clinical data and DRGs (Fig. 3D,E). Our findings revealed a negative correlation between AC012615.1 and the majority of DRGs, whereas the remaining four DRlncRNAs exhibited a positive correlation with most DRGs (Fig. 3E). Following that, the disulfidptosis-related lncRNA-based signature (DRLS) score of each patient was computed applying the formula below: DRLS Score (Risk Score) = 0.152323368591861 × expr AL365181.2 + 0.228911037835446 × expr GSEC + 0.203628306081756 × expr AC093673.1 + (− 0.44972537859839) × expr AC012615.1 + 0.292888245903172 × expr AL606834.1.

The patient cohort was stratified into high-risk and low-risk groups according to the optimal cut-off value of DRLS score. Subsequently, K–M analysis was performed among the training set, and the results revealed a significant improvement in OS of LUAD patients with low DRLS score compared to those with high DRLS score (Fig. 3F, P < 0.001). Further investigation involved assessing the distribution of survival times, survival statuses, and risk scores between the two groups. Simultaneously, the expression profiles of five DRlncRNAs were compared within these two sets (Fig. 3G). Our results highlighted remarkably increased expression levels of AL365181.2, GSEC, AC093673.1, and AL606834.1 in the high-risk group, consistent with their respective positive correlation coefficients in the DRLS score calculation formula, indicating their role as risk factors. Additionally, in the time-based ROC curves, the area under the curve (AUC) values of 1, 3, and 5 years were 0.701, 0.668, and 0.618, respectively. As for the combined ROC curves, the AUC values considering clinical features were 0.668 (Risk), 0.658 (Stage), 0.523 (Gender), and 0.519 (Age) (Fig. 3H). Ultimately, the t-SNE and PCA were utilized to reduce dimensionality and visualize the distinct characteristics between the two groups. The results conclusively showed significant and consistent differences in the distributions of the high-DRLS and low-DRLS groups (Fig. 3I).

### Evaluation of the DRLS model

**Figure 4:**
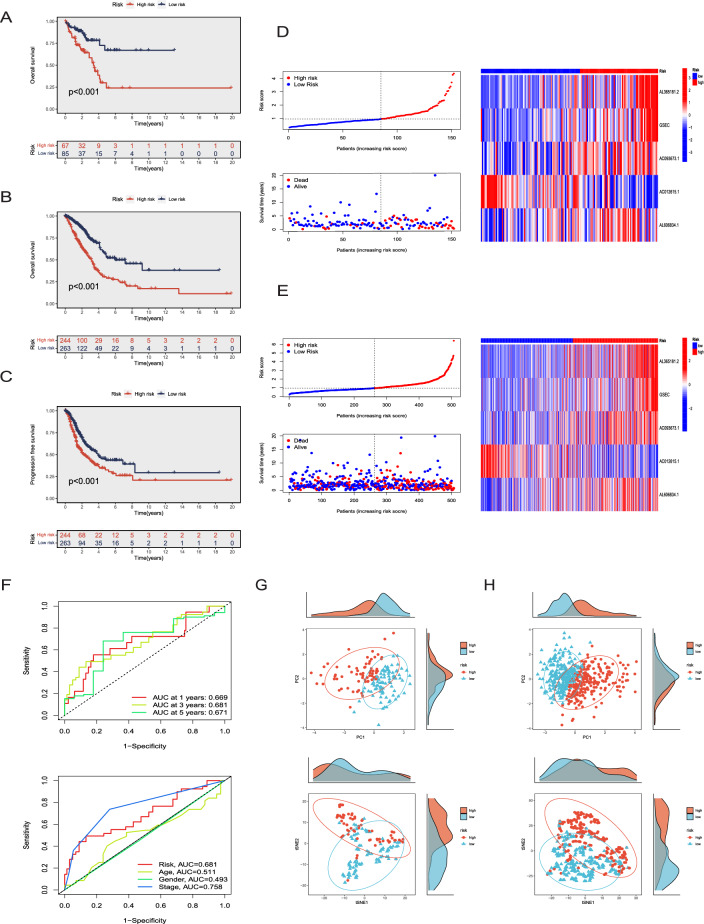
Evaluation of the DRLS model. (**A,B**) K–M analysis for the testing set and the entire set. (**C**) The PFS curve was constructed for the entire set. (**D,E**) The distribution of DRLS scores as well as status and time of survival for each LUAD patient, along with the clustering heatmap of the 5 DRlncRNAs in the two sets. (**F**) ROC curves for predicting the OS of 1, 3, and 5-years, along with clinical ROC curves in the two sets. (**G,H**) PCA and t-SNE were performed on LUAD patients in the two sets.

We further examined the reliability and predictive capacity of established DRLS by calculating DRLS scores (risk scores) for LUAD patients among the testing and entire sets utilizing the same computational formula. Similar to the training set, patients in the two sets were stratified into high and low risk groups. Subsequent K–M analyses were conducted among the two sets, and the findings consistently revealed the low-risk groups displayed markedly superior OS compared to the high-risk groups (Fig. 4A,B, P < 0.001). Additionally, the progression-free survival (PFS) was demonstrated notably enhanced in the groups with low DRLS score in comparison to the high-DRLS groups (P < 0.001) among the entire set. An evaluation for the distribution of survival time and survival status, based on DRLS scores, was also conducted in the two groups among both the testing and entire sets. The expression profiles of these 5 DRlncRNAs in the two groups displayed consistent outcomes with those observed in the training sets (Fig. 4D,E). Subsequently, ROC curves of the two sets reflected the same trends observed in the training sets. As for the AUC values at 1, 3, and 5 years in the testing set were 0.669, 0.681, and 0.671, respectively. While the corresponding AUC values were 0.690, 0.671, and 0.631 within the entire set (Fig. 4F). Furthermore, PCA and t-SNE were also applied in the two sets to visualize the sample distributions of high-DRLS and low-DRLS groups (Fig. 4G,H). These findings implied the DRLS based on 5 DRlncRNAs could consistently and accurately predict the outcomes for LUAD patients with a high level of dependability.

### Establishment and validation of a nomogram combined with clinical characteristics

**Figure 5:**
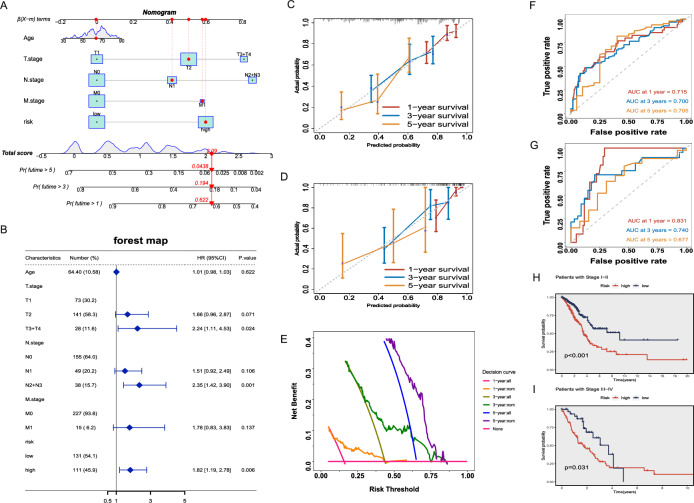
Establishment and validation of a nomogram combined with clinical characteristics. (**A**) A nomogram for OS prediction of LUAD patients at 1, 3, and 5-years, constructed by integrating DRLS, age, and TNM stage in the training set. (**B**) T stage, N stage, and risk score as the major prognostic factors of the nomogram in a forest plot. (**C,D**) Calibration curves based on the nomogram in the training (**C**) and testing (**D**) sets. (**E**) DCA curves of the nomogram for survival prediction among LUAD patients. (**F,G**) Time-dependent ROC curves based on the nomogram in the training (**F**) and testing (**G**) sets. (**H,I**) Kaplan–Meier survival analyses for patients with Stage I–II (**H**) and Stage III–IV (**I**).

Considering the widespread clinical application of TNM staging and the reliable predictive ability of DRLS, we constructed a nomogram through combining TNM staging with DRLS in the training set. Subsequently, this nomogram was used to compute scores of each patient, enabling better prediction of their 1, 3, and 5-year prognoses (Fig. 5A). We also generated a forest plot based on the nomogram, which highlighted T stage, N stage, and risk score as the primary prognostic factors (P < 0.05, Fig. 5B). Next, we plotted calibration curves to further assess the predictive accuracy of this nomogram among the training and testing sets. These curves illustrate a concordance between the observed and predicted values (Fig. 5C,D). DCA was employed to assess the utility and effectiveness of this prediction model. The findings indicated the potential of this nomogram for accurate prediction of the LUAD patients’ survival probabilities at various time intervals. (Fig. 5E). Furthermore, time-dependent ROC curves were constructed using this nomogram among the training and testing sets. In the training set, the AUC values for 1, 3, and 5 years were 0.715, 0.7, and 0.705, respectively (Fig. 5F). While in the testing set, the corresponding AUC values were 0.831, 0.74, and 0.677 (Fig. 5G). In summary, this nomogram demonstrated the ability for accurate prognosis prediction and the potential in refining clinical treatment decisions for LUAD patients. Finally, patients were stratified into early-stage (I–II) and advanced-stage (III–IV) groups, and K–M analysis was applied for validating the disparities in survival of the high and low-risk groups among patients in each stage. We found that regardless of the stage, LUAD Patients categorized within the low-risk group exhibited notably superior survival rates in comparison to those in the high-risk group (P < 0.05, Fig. 5H,I).

### Underlying molecular mechanisms of the DRLS

An investigation employing 513 DEGs that differentiated between the high-risk and low-risk groups (|Log2FC| > 1.0, P-value < 0.05) were conducted to unravel the underlying molecular mechanisms responsible for the marked distinctions observed in our multifaceted analyses between the two groups (Table S5). The GO analysis unveiled significant enrichment of these DEGs in cellular components (e.g., cytoplasmic region and plasma membrane bounded cell projection cytoplasm) and molecular functions (e.g., oxidoreductase activity and serine-type endopeptidase inhibitor activity) (Supplementary Fig. S1A). Furthermore, the KEGG analysis revealed a predominant association of these DEGs with pathways pertinent to biological process of tumors, such as the PI3K-Akt signaling pathway, and pathways linked to metabolic processes, including arachidonic acid metabolism (Supplementary Fig. S1B). Subsequently, we explored the differences in pathways enriched with DEGs in the low- and high-risk groups through GSVA analysis. Notably, we observed significant enrichment of DEGs in the high-risk group across a spectrum of metabolic pathways, encompassing glycolysis gluconeogenesis, pentose phosphate pathway, and galactose metabolism. Furthermore, these DEGs exhibited enrichment in pathways associated with p53 signaling, cell cycle, and DNA replication, which could potentially promote tumor progression in the high-risk cohort (Supplementary Fig. S1C).

Subsequently, correlation analysis was performed to examine the association between five DRlncRNAs employed in constructing the DRLS and the risk score with regard to tumor-related pathways. Our analysis revealed positive correlations between AC093673.1, AC606834.1, and the risk score with tumor-related pathways. Conversely, AC012615.1 exhibited negative associations with all tumor-related pathways, thereby reinforcing our earlier conclusion that AC012615.1 functions as a protective factor (Supplementary Fig. S1D). Furthermore, the results of GSEA revealed notable pathway enrichments in the high-DRLS group, including cell cycle, focal adhesion, metabolism of xenobiotics by cytochrome P450, the P53 signaling pathway, and steroid hormone biosynthesis (Supplementary Fig. S1E). On the contrary, the low-risk group demonstrated significant pathway enrichments in areas such as allograft rejection, asthma, and the immune network for IgA production (Supplementary Fig. S1F). In summary, the DEGs identified between the two groups with distinct risk score may exert a significant influence on the pathogenesis of LUAD.

### Somatic mutation landscape

**Figure 6:**
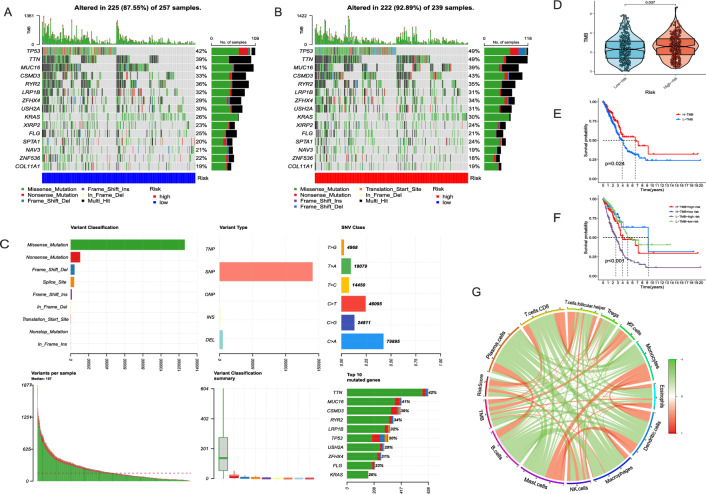
Somatic mutation landscape. (**A,B**) The mutation landscapes of the top 15 genes in the low (**A**) and high-risk (**B**) groups, ranked by mutation frequency. (**C**) The visual representation of mutation details was provided. The top 10 mutated genes in LUAD were illustrated in a horizontal bar graph. (**D**) The TMB distinctions of the two risk groups were displayed in a violin plot. (**E,F**) Survival analysis for LUAD patients based on the TMB (**E**) and the integration of risk scores and TMB (**F**). (**G**) The relationships between risk scores, TMB, and immune cell infiltration were depicted in a correlation chord diagram.

Tumor mutational burden (TMB) was the measure used to assess mutation frequency within tumor cells, and we conducted an analysis of somatic mutations of the high and low risk groups. This analysis unveiled 15 genes with the highest mutation frequencies in both groups, including TP53, TTN, MUC16, CSMD3, RYR2, LRP1B, ZFHX4, USH2A, and KRAS (Fig. 6A,B). The specifics of these mutations are visually presented in Fig. 6C. The most prevalent mutation type was missense mutations, characterized by a greater frequency of single nucleotide polymorphism (SNP) compared to insertions (INS) and deletions (DEL). Unlike C > T and C > G transitions, C > A mutations emerged as more frequent. The top 10 mutated genes of LUAD were displayed in the horizontal bar graph. Additionally, a quantitative assessment of TMB indicated the patients with high-DRLS scores displayed notably elevated TMB scores in comparison to those with low-risk scores (Fig. 6D, P < 0.05). To investigated the impact of TMB on prognosis, whole LUAD patients were categorized into high and low TMB groups, with the median TMB score serving as the cutoff point. The results of K–M analysis revealed the patients with high TMB scores exhibited improved OS in comparison to those with low TMB scores (Fig. 6E, P < 0.05). Interestingly, our subsequent analysis, which sought to predict the outcomes of LUAD patients by combining TMB level and risk scores, revealed that patients with low-risk score and high TMB score exhibited markedly improved prognoses in comparison to patients in all other groups. This observation suggested the risk score surpassed TMB in predicting individual outcomes (Fig. 6F, P < 0.001). Finally, we examined the relation between the risk score, TMB, and immune cell infiltration, visually representing the results through a correlation chord diagram (Fig. 6G).

### Immune cell infiltration landscape

**Figure 7:**
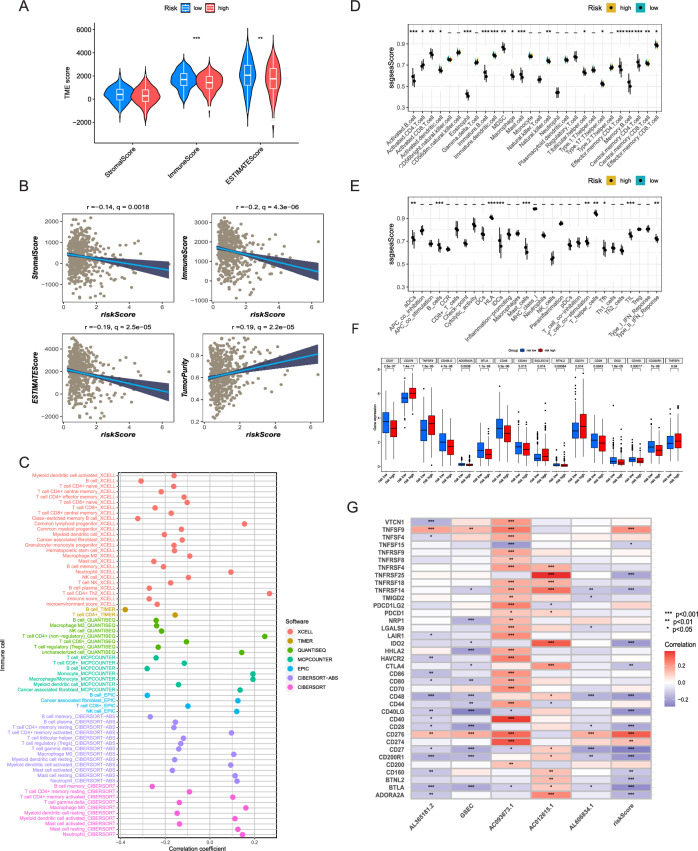
Immune cell infiltration landscape. (**A**) Differences in Stromal Score, Immune Score, and ESTIMATE Score between the high and low risk groups were portrayed in violin plots. (**B**) The correlation between the risk score and the three scores were depicted in the scatter plots. (**C**) The relationship between risk scores and tumor-infiltrating immune cells was evaluated through correlation analysis employing 7 algorithms. (**D,E**) ssGSEA scores were calculated for immune cells and immune function in both risk groups. (**F**) Prevalent immune checkpoint markers were assessed for differential expression among various risk groups. (**G**) The correlation of the 5 DRlncRNAs, risk score, and 37 immune checkpoint markers. ***P ≤ 0.001, **P ≤ 0.01, and *P ≤ 0.05.

Over the past few decades, a large number of studies has highlighted the predominance of TME in the development and progression of various types of tumors. Immune cells, as essential components of the TME, engage in complex interactions and regulatory processes with tumor cells, subsequently influencing tumor progression and treatment outcomes. Therefore, the immune score, stromal score, and ESTIMATE score were computed for each patient employing the ESTIMATE algorithm, with the aim of exploring potential distinctions in immune cell infiltration within the high- and low-risk groups. The results indicated an increased level of immune cell infiltration within the low-risk group, characterized by significantly improved immune and ESTIMATE scores when compared to those of the high-risk group (Fig. 7A). In correlation analysis, it was observed that the risk score exhibited a negative correlation with the immune score, stromal score, and ESTIMATE score, while positively related to tumor purity (Fig. 7B). Following this, the association of risk score and tumor-infiltrating immune cells was investigated using 7 distinct algorithms, in order to ensure a more dependable assessment (Fig. 7C). Our findings indicated that infiltration levels of diverse immune cell types, including CD8+ T cells, B cells, NK cells, macrophages, and mast cells, exhibiting an inverse correlation with the risk score. This observation suggested the possibility of increased immune cell infiltration within the TME of patients categorized by low-DRLS score. Next, the ssGSEA algorithm was utilized for evaluating the immune status of TME in the two groups with distinct risk scores. Our results implied the low-risk group manifested higher ssGSEA scores in diverse immune cells, such as activated CD8 T cells, mast cells, NK cells, and effector memory CD4 T cells, in comparison to the high-risk group (Fig. 7D, P < 0.05). Additionally, the enhanced immune function scores were observed among the low-risk group, such as activated Dendritic Cells, B cells, Human Leukocyte Antigen, inactive Dendritic Cells. These findings suggested that patients categorized by low DRLS exhibited a more proactive immune response against tumor progression, which in turn may lead to a more favorable outcome (Fig. 7E, P< 0.05). Furthermore, the evaluation of 50 immune checkpoints (ICs) expressions was conducted within the two groups with different level of risk score (Fig. 7F). We identified differential expression in 18 ICs within the two groups (P < 0.05). Notably, immune checkpoints such as CD27, CD28, CD48, CD160, CD244, CD40LG, ADORA2A, BTLA, BTNL2, IDO2, and CD200R1 exhibited significantly higher expression levels in patients of the low-risk group. This suggests that patients categorized by low DRLS scores may potentially benefit from immunotherapies targeting these specific ICs. Finally, analyses were conducted for exploring the correlation of 5 DRlncRNAs and risk scores with 38 immune checkpoints. The findings revealed a tightly positive correlation between AC093673.1 and AC012615.1 with most ICs, while AL365181.2, GSEC, AL606834.1, and risk scores exhibited a negative correlation with the most ICs (Fig. 7G). These findings indicated the elevated levels of immune infiltration within low-risk group, which could potentially contribute to an enhanced prognosis. Additionally, patients with elevated expression levels of most ICs demonstrated the potential to benefit from specific immunotherapies.

### Estimation of immunotherapy response and drug sensitivity

**Figure 8:**
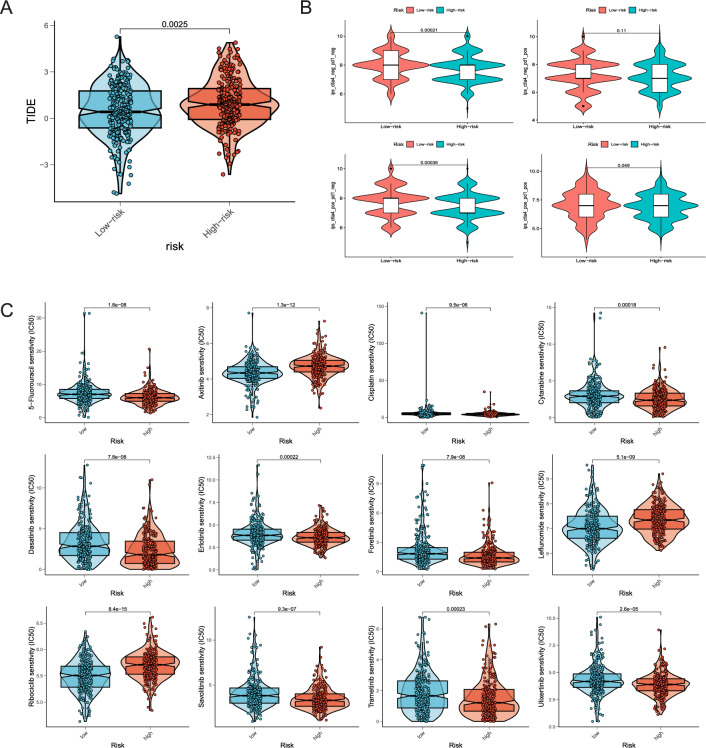
Estimation of immunotherapy response and drug sensitivity. (**A,B**) The TIDE scores and IPS scores of the high and low risk groups were observed in the violin plots respectively. (**C**) The IC50 values for chemotherapy and targeted drugs in the two groups were displayed.

The TIDE website was utilized to assess tumor immune evasion and the impact of immunotherapy on both the low- and high-risk groups. The considerably higher scores were observed in the high-risk group, suggesting that patients characterized by high DRLS score could be more susceptible to immune evasion, potentially resulting in a less favorable response to immunotherapy (Fig. 8A). Following this, we obtained Immunophenoscore (IPS) for LUAD patients from the TCIA database with the aim of evaluating the predictive value of DRLS in the context of immunotherapy. This assessment aimed to estimate patients’ immunogenicity, predicting their potential responsiveness to immune checkpoint blockades (ICBs). The findings showed elevated IPS scores in the low-risk group, suggesting the potential of patients characterized by low DRLS scores might more favorably respond to ICBs (Fig. 8B). Given that chemotherapy and targeted therapies are standard treatments for intermediate and advanced stage LUAD, we conducted an additional investigation to assess how patients categorized by DRLS respond to commonly used antitumor agents. Interestingly, when comparing the IC50 values of these agents between the two groups, a significant improvement was observed in response to targeted therapies (e.g., Dasatinib, Erlotinib, Oretinib, Savolitinib, Trametinib, and Ulixertinib) and chemotherapeutic treatments (e.g., 5-Fluorouracil, Cisplatin, and Cytarabine) in the low-risk group. These findings indicated that patients characterized by elevated risk scores might respond more favorably to these drugs (Fig. 8C).

### Identification of molecular subtypes

**Figure 9:**
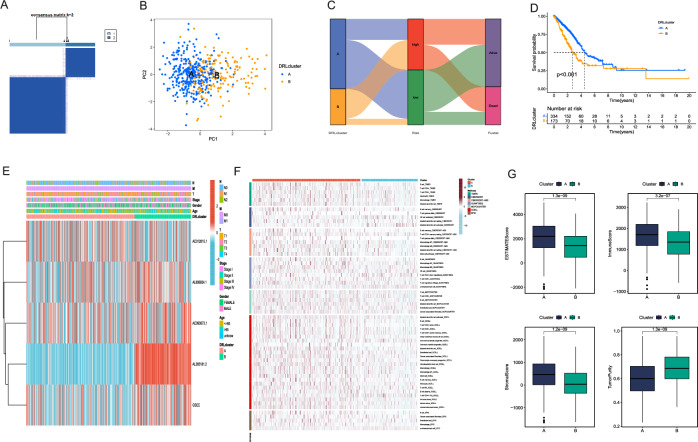
Identification of molecular subtypes based on the expression of 5 DRlncRNAs. (**A**) All LUAD patients were classified into two clusters (k = 2). (**B**) The distribution of two clusters were depicted using PCA. (**C**) The correlation of two clusters and the groups with distinct risk scores was visualized in a Sankey plot. (**D**) K–M analysis for the OS prediction of the two clusters. (**E**) Clinical information integrated with clustering results were visualized in a clinical correlation heatmap. (**F**) A heatmap was used to illustrate variations in immune cell infiltration using 7 different algorithms. (**G**) The differences in stromal score, immune score, ESTIMATE score, and tumor purity between the two clusters were presented in box plots.

We employed an unsupervised consensus clustering method to investigate distinct molecular subtypes according to the DRlncRNAs expression. Two clusters were generated for patients with LUAD, and we confirmed an optimal cluster stability at k = 2 (Fig. 9A). We employed PCA to evaluate the sample distribution within cluster A and cluster B (Fig. 9B). Subsequently, we explored the associations between the clusters and two groups. The findings revealed that cluster A primarily included patients categorized to the low-risk group, whereas cluster B was predominantly composed of patients characterized with high-risk score (Fig. 9C). Subsequent survival analysis indicated a significantly better prognosis of cluster A in comparison to cluster B (Fig. 9D). We generated a clinically relevant heat map by integrating clinical data, the expression levels of 5 DRlncRNAs, and the clustering results (Fig. 9E). Similarly, we assessed immune cell infiltration within the two clusters using 7 algorithms utilized in previous investigation. Our findings revealed higher levels of immune cell infiltration, such as B cells and CD4+ T cells within the TME of cluster A (Fig. 9F). This implied that the TME of cluster A recruited a greater number of immune cells and initiated a more adaptive immune response, thereby fostering an inflammatory tumor microenvironment. Analyzing the TME scores for the various clusters revealed the enhanced immune scores, stromal scores, and ESTIMATE scores among the cluster A when compared to cluster B. Nonetheless, cluster A exhibited lower tumor purity than cluster B (Fig. 9G).

### Validation of GSEC expression and biological function in LUAD

**Figure 10:**
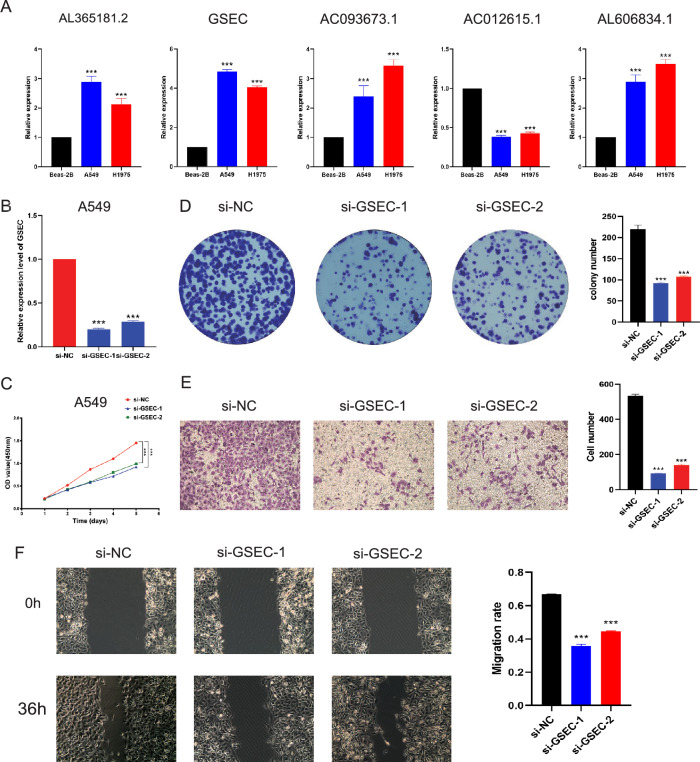
The validation of GSEC expression and its biological function in LUAD. (**A**) Expression levels of the 5 DRlncRNAs were evaluated between BEAS-2B, NCI-H1975 and A549 cells using RT-qPCR. (**B**) The knockdown efficiency of GSEC in the A549 cell line, validated using RT-qPCR. The CCK-8 assay (**C**) and colony formation assay (**D**) were applied to determine the influence GSEC knockdown on LUAD cell proliferation. The transwell assay (**E**) and the wound healing assay (**F**) were adopted to explore the migration of LUAD cells upon GSEC knockdown.

In this study, we assessed the expression of five DRlncRNAs, which were utilized in our model construction, in LUAD cell lines including A549, H1975, and BEAS-2B using RT-qPCR (Fig. 10A). Among these, four DRlncRNAs exhibited upregulation in both LUAD cell lines (AL365181.2, GSEC, AC093673.1, AL606834.1), while AC012615.1 showed significant downregulation in both LUAD cell lines. According to our research, GSEC is one of the four highly expressed DRlncRNAs in tumor samples and related to a relatively higher hazard ratio (HR 1.257), indicating an unfavorable prognosis for LUAD patients. Furthermore, we observed an association between GSEC and ferroptosis, cuproptosis and disulfidptosis. Consequently, we conducted in vitro experiments for investigating the role of GSEC in LUAD progression. Firstly, GSEC was effectively silenced in A549 cells (Fig. 10B). Subsequently, we evaluated the impact of GSEC downregulation on LUAD cell proliferation through CCK-8 experiments. We found that the proliferation capacity of A549 cells decreased upon GSEC knockdown in comparison to the control group (Fig. 10C), implying that GSEC may promote LUAD cell proliferation. Similarly, colony formation experiments demonstrated a marked reduction in the number of colonies within GSEC downregulation group in comparison to the control group (Fig. 10D). Transwell assays also implied that GSEC knockdown significantly inhibited the migration of A549 cells (Fig. 10E). Wound healing experiments revealed that GSEC downregulation significantly delayed wound healing (Fig. 10F). These results implied the involvement of GSEC in the proliferation and migration of LUAD cells, highlighting its potential in promoting LUAD progression.

## Discussion

With the high morbidity and mortality, lung cancers threat human life at a high extent^31^, especially for LUAD, the most common pathological type of NSCLC. Surgical resection continued to be the optimal approach for the majority of early-stage LUAD patients, while some patients with advanced-stage (IV) LUAD exhibited improved prognostic outcomes with treatments such as immunotherapy^32^. Nevertheless, the treatment effects and prognosis of the majority of advanced LUAD patients generally remain to be unsatisfied. As a consequence, the exploration of new prognostic biomarkers and novel therapeutic agents for LUAD remain to be crucial and urgent. In past decades, increasing research revealed lncRNAs were involved in variety of key biological processes of LUAD with profound influence on progression and treatment of LUAD^33^. The induction of RCD including apoptosis, pyroptosis, necroptosis, ferroptosis and cuproptosis proved to be a promising anti-tumor mechanism^5,6^. It has been demonstrated that lncRNAs could affect the progression and sensitivity to therapy of lung cancer via regulation of RCD. For example, lncRNA LINC00336 highly expressed in lung cancer and inhibited ferroptosis via binding to the RNA-binding protein ELAVL1^34^. In addition to ferroptosis, cuproptosis is another key point of RCD research. Furthermore, several cuproptosis-related lncRNA-based signatures have been developed for the prognosis prediction of LUAD patients^35,36^.

Disulfidptosis, distinct from established forms of regulated cell death (RCD) such as ferroptosis and cuproptosis, represents a recently reported and novel subtype of RCD. This discovery opens up new avenues for exploring potential therapeutic strategies in cancer treatment. Liu’s research revealed that cancer cells overexpressing SLC7A11 undergo a unique form of cell death when exposed to glucose deprivation, distinct from recognized cell death subtypes. Conventional drug-based approaches for inhibiting cell death and knockdown of essential genes associated with ferroptosis or apoptosis do not impede this novel form of cell death. Instead, thiol oxidizing agents like diamide and diethyl maleate significantly enhance this form of cell demise. Hence, this unique cell death phenomenon was denoted as “disulfidptosis”^8^. However, disulfidptosis has not been comprehensively studied in lung cancer, meanwhile there is no research exploring the correlation between disulfidptosis, lncRNAs and LUAD. Therefore, our study developed a disulfidptosis-related LncRNA-based signature (DRLS) to predict the prognosis of LUAD as well as to explore its relationship with TIME and immunotherapy response.

In the initial phase, we screened all the lncRNAs correlated to 16 genes which reported to be associated with disulfidptosis and further developed a prognostic prediction signature, referred to as DRLS, with the aim of predicting the prognosis of LUAD patients, using transcriptome data and clinical information of LUAD acquired from TCGA (Fig. 2). The signature comprised 5 disulfidptosis-related lncRNAs (DRlncRNAs): AL365181.2, GSEC, AC093673.1, AC012615.1, and AL606834.1, which were identified and validated through a screening process involving LASSO, univariate and multivariate Cox regression analyses among the training, testing as well as entire sets. Of these DRlncRNAs, AL365181.2, GSEC, AC093673.1, and AL606834.1 were determined as prognostic risk factors, whereas AC012615.1 was identified be a protective factor. GSEC, one of the DRlncRNAs, has previously been indicated as an oncogene in diverse tumors. In the case of LUAD, GSEC played an active role in the promotion of tumor progression through the GSEC/miRNA-101-3p/CISD1 Axis and showed a significant association with ferroptosis, as confirmed by previous research^37^. Additionally, several research reported GSEC could promote the malignant process of triple-negative breast cancer and hepatocellular carcinoma via the GSEC/miR-202-5p/AXL axis and the GSEC/miR-101-3p/SNX16/PAPOLG axis^38,39^, respectively. Furthermore, lncRNA AL606834.1 has been used for prognostic models of lung adenocarcinoma and pancreatic cancer^40,41^, AL365181.2 has been applied in multiple prognostic models of lung adenocarcinoma^42,43^, and AC012615.1 has been studied in a prognostic model of glioblastoma^44^. These findings provided the robust evidence for the construction of our DRLS score based on these 5 lncRNAs. Subsequently, risk scores for each patient were calculated using our signature and enabled the classification of LUAD patients categorized by this score. The DRLS score exhibited the reliable predictive capacity for prognosis of LUAD patients both in the training, testing and entire sets through a series of analyses such as survival analysis, ROC analysis and so on (Figs. 3, 4).

TMB takes a pivotal part in the clinical response of tumors to immunotherapy^45,46^. Elevated TMB levels may result in increased effectiveness of ICIs. The results demonstrated heightened mutation frequencies in TP53, TTN, and MUC16 within the high and low risk groups (Fig. 6). TP53, functioning as a transcription factor, was involved in various cancer-suppressive biological processes, including apoptosis and DNA damage repair^47–49^. In the survival analysis, patients characterized by high TMB and low risk scores exhibited the most favorable prognosis. Future studies should aim to delve deeper into whether the enhanced prognosis can be attributed to an increased occurrence of mutations in genes such as TP53 and TTN.

The tumor immune microenvironment (TIME) constitutes a pivotal element within the tumor microenvironment (TME), exerting a substantial impact on the biological processes of tumors and the outcomes of anticancer therapies. As a result, an evaluation of disparities in the TIME between high and low-risk groups was undertaken using eight distinct algorithms (Fig. 7). This analysis demonstrated the risk score negatively correlated with the infiltration level within the most immune cells, indicating an elevated presence of immune cell infiltration in the TME of patients within the low-risk group. Immunotherapy, which mostly relies on ICIs, has exhibited notable improvements in prognostic outcomes for advanced cancer patients^50,51^. In our research, several ICs displayed differential expression within the high and low risk groups (Fig. 7). Notably, we found the significantly elevated expression levels of numerous ICs among patients with low DRLS scores, such as Cluster of differentiation 27 (CD27) and CD28 which significantly involved in T-cell activation and diverse T-cell processes, indicating the probability of these patients for more favorable immune responses^52,53^. In addition, IPS and TIDE scores were calculated to estimate the immunogenicity and immune evasion probability of each patient in two groups with distinct risk score levels (Fig. 8). Our results implied the potential for more favorable immunotherapy responses in patients with lower DRLS score. Since chemotherapy and targeted therapy are conventional therapeutic strategies for intermediate and advanced LUAD, we further determined the responses of low- and high-risk groups to common antitumor agents. Our results showed the IC50 values of targeted therapeutic agents such as Dasatinib, Erlotinib, oretinib, Savolitinib, Trametinib and Ulixertinib and chemotherapeutic agents such as 5-Fluorouracil, Cisplatin and Cytarabine exhibited a notable increase in patients with low DRLS scores, implying that these patients may display a more favorable treatment response to these drugs.

In our investigation, we observed a substantial upregulation of GSEC expression in tumor cells, and identified its correlation with ferroptosis, cuproptosis, and disulfidptosis. Consequently, we selected GSEC for subsequent experimental validation. During the in vitro experiments, we conducted CCK8, colony formation, transwell, and wound-healing assays, and ultimately demonstrated that downregulating GSEC inhibited LUAD cell proliferation and invasion. Our findings implied that GSEC could potentially serve as a novel therapeutic target for LUAD patients.

Certainly, certain limitations were encountered within the study, as is often the case. Firstly, this study mainly used bioinformatics analysis, and the conclusions need further experimental validation. Secondly, our cellular experiments remained insufficient as we only preliminarily explored the functional phenotype of GSEC in LUAD cells, therefore in vivo experiments could be performed in the future for further validation. Thirdly, the molecular interactions between lncRNAs and disulfidptosis-related genes need to be further characterized. Finally, our data were derived from the TCGA database and lacked external dataset validation, and there were some bias caused by the relatively small sample size. In summary, an independent prognostic prediction for LUAD patients can be made through the utilization of the DRlncRNAs signature, potentially offering valuable insights for the immunotherapeutic management of LUAD patients.

## Conclusions

An extensive bioinformatics analysis was conducted to develop a prognostic model for assessing the prognosis of patients with LUAD. This model relies on the identification of five DRLncRNAs. This model, referred to as the disulfidptosis-related lncRNA-based signature (DRLS), is expected to facilitate a deeper understanding of the roles played by disulfidptosis and lncRNAs in LUAD, with the potential to enhance the prognosis of LUAD patients.

### Supplementary Information

Supplementary Information.

## Data Availability

RNA-sequencing data and clinical data of LUAD were downloaded from The Cancer Genome Atlas (TCGA) (https://portal.gdc.cancer.gov/). Further inquiries can be directed to the corresponding author.
