# Supplementary material for: A disulfidptosis-related lncRNA signature for predicting prognosis and evaluating the tumor immune microenvironment of lung adenocarcinoma
Source: Sci Rep. 2024 Feb 26;14:4621. doi: 10.1038/s41598-024-55201-7 (PMC10897395; doi:10.1038/s41598-024-55201-7)
Supplement: Supplementary file 1 — Supplementary Information. [file 41598_2024_55201_MOESM1_ESM.pdf]

A disulfidptosis-related lncRNA signature for predicting prognosis and evaluating the tumor immune microenvironment of lung adenocarcinoma

Supplementary figures:

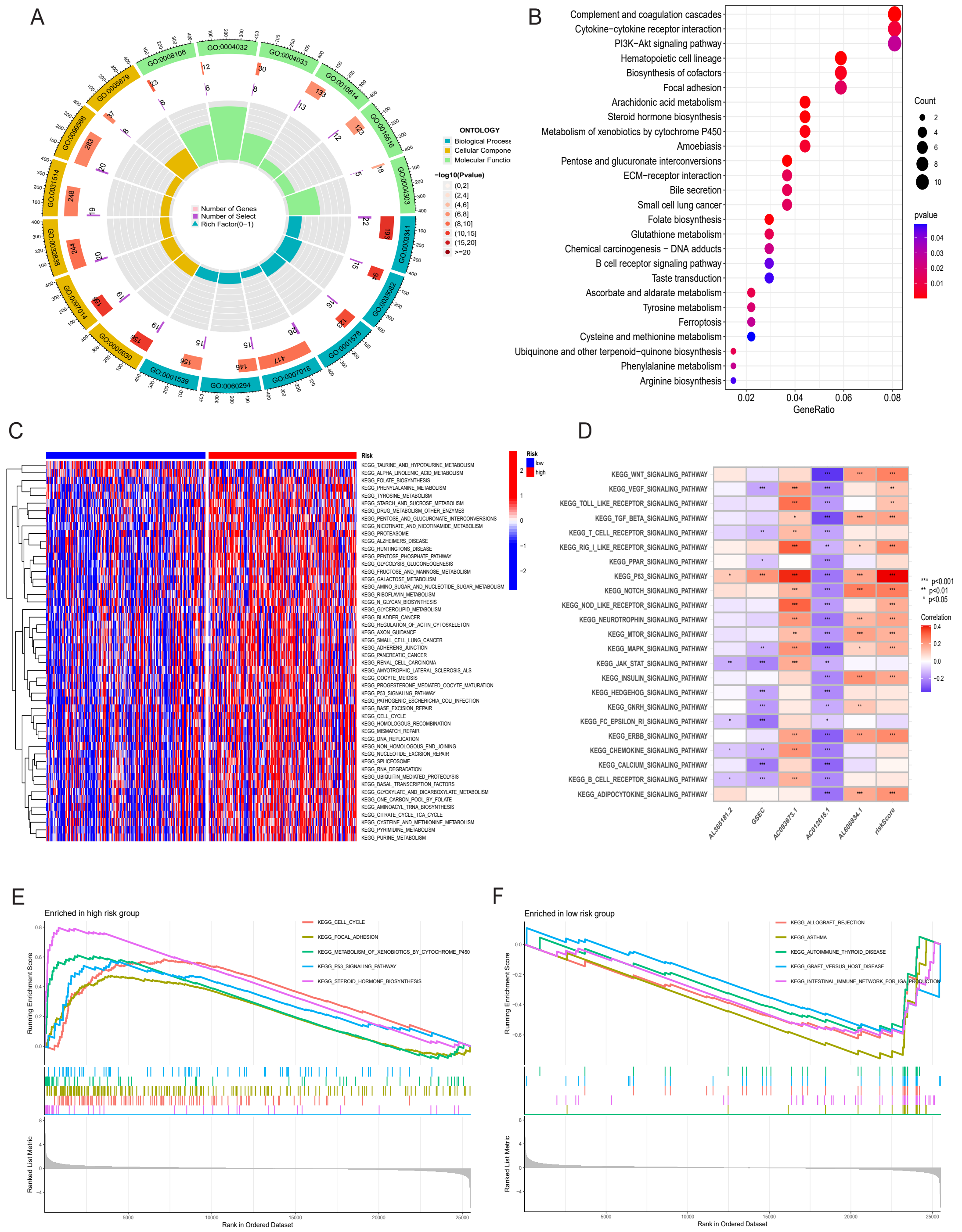

Supplementary Fig. 1. Underlying molecular mechanisms of the DRLS.

(A) The results of the GO analysis for DEGs. (B) Pathways obtained from the KEGG analysis were visualized in a bubble plot. (C) The GSVA results were presented in a heatmap. (D) The results of correlations analysis for the 5 DRlncRNAs, risk score, and different pathways. (E, F) GSEA analysis for the top 5 enriched pathways in the high (E) and low DRLS group (F).

Supplementary tables:

Table S1. 16 disulfidptosis-related genes (DRGs).

ACTB
FLNA
FLNB
GYS1
LRPPRC
MYH9
NCKAP1
NDUFA11
NDUFS1
NUBPL
OXSM
PRDX1
RPN1
SLC3A2
SLC7A11
TLN1

Table S2. 104 disulfidptosis-related LncRNAs in LUAD were identified by the use of Pearson's correlation analysis.

AC020558.2	LMNTD2-AS1	AL928654.2
AC003086.1	AC087741.1	AC074212.1
AL645939.5	AL355353.1	AC010331.1
AP006621.4	AP003119.2	AC006033.2
AL365181.2	AL136295.2	LYRM4-AS1
AC133552.5	AL132800.1	AC011498.6
STARD7-AS1	AC026785.3	AC011815.1
AC104472.4	C10orf55	LINP1
AC016773.1	AC145207.8	MIR4713HG
AC137695.3	SNHG16	CAHM
LINC01023	AC234917.3	AL606834.1
AL139039.3	FGD5-AS1	SNHG10
SNHG12	AC092115.3	AP003419.3
AC015922.2	ALMS1-IT1	AC084125.2
AL139353.2	PTPRN2-AS1	GAS5
AC112220.2	LINC01269	C9orf163
LINC01311	AC004943.2	AC091057.1
AC116407.2	MANCR	AL606489.1
AC012073.1	AC087289.2	AC008608.2
AC114730.3	KMT2E-AS1	OIP5-AS1
AP001412.1	USP46-DT	SNHG11
MHENCR	NALT1	AL513320.1
AC011462.4	ARRDC1-AS1	AC090559.1
CACTIN-AS1	AC132872.2	AL513165.1
AC027601.1	KTN1-AS1	AC005387.1
AC092718.4	NORAD	AC015912.3
AP001189.3	PP7080	
MIR762HG	AC009065.4	
AC092171.4	AC106045.1	
GSEC	AC012615.1	
STK4-AS1	LINC01063	
AL365181.3	LINC01960	
AL390719.2	RNASEH1-AS1	
AL590666.2	AC008760.1	
AL033527.2	LINC01089	
AL450326.1	TMEM147-AS1	
ENTPD3-AS1	EMSLR	
AC008764.6	PTOV1-AS2	
AC093673.1	AC021037.1	

Table S3. The clinical and pathological characteristics of LUAD samples between training and testing set.

Covariates	Type	Total	Test	Train	P value
Age	<=65	239(47.14%)	82(53.95%)	157(44.23%)	0.0828
	>65	258(50.89%)	69(45.39%)	189(53.24%)	
	unknow	10(1.97%)	1(0.66%)	9(2.54%)	
Gender	FEMALE	272(53.65%)	81(53.29%)	191(53.8%)	0.9928
	MALE	235(46.35%)	71(46.71%)	164(46.2%)	
Stage	Stage I	272(53.65%)	89(58.55%)	183(51.55%)	0.3128
	Stage II	120(23.67%)	35(23.03%)	85(23.94%)	
	Stage III	81(15.98%)	18(11.84%)	63(17.75%)	
	Stage IV	26(5.13%)	9(5.92%)	17(4.79%)	
	unknow	8(1.58%)	1(0.66%)	7(1.97%)	
T	T1	169(33.33%)	55(36.18%)	114(32.11%)	0.747
	T2	271(53.45%)	80(52.63%)	191(53.8%)	
	T3	45(8.88%)	11(7.24%)	34(9.58%)	
	T4	19(3.75%)	6(3.95%)	13(3.66%)	
	unknow	3(0.59%)	0(0%)	3(0.85%)	
M	M0	338(66.67%)	96(63.16%)	242(68.17%)	0.562
	M1	25(4.93%)	9(5.92%)	16(4.51%)	
	unknow	144(28.4%)	47(30.92%)	97(27.32%)	
N	N0	327(64.5%)	105(69.08%)	222(62.54%)	0.3821
	N1	95(18.74%)	27(17.76%)	68(19.15%)	
	N2	71(14%)	16(10.53%)	55(15.49%)	
	N3	2(0.39%)	1(0.66%)	1(0.28%)	
	unknow	12(2.37%)	3(1.97%)	9(2.54%)	

Table S4. Multivariate Cox results of lncRNAs based on TCGA data

LncRNA	Coef	HR
AL365181.2	0.152323369	1.16453675
GSEC	0.228911038	1.257230188
AC093673.1	0.203628306	1.225842431
AC012615.1	-0.449725379	0.637803282
AL606834.1	0.292888246	1.340292999

Table S5. 513 DEGs between high-risk and low-risk groups

gene	lowMean	highMean	logFC	pValue	fdr
AC113349.1	1.628014132	0.534667486	-1.60639937	0.000250838	0.00096306
LAMA1	0.747782826	2.742797883	1.87495707	0.004829375	0.011632306
CYP4F11	5.191412421	18.94356161	1.867508591	2.21E-07	2.57E-06
FAM133A	2.080410646	8.287253484	1.994025725	0.011075449	0.023335379
G6PD	93.2340294	232.9054501	1.320815876	3.19E-16	8.73E-14
RSPO3	2.889194106	9.404611612	1.702701233	0.007063773	0.015977273
RPL13AP17	2.312860646	0.90218627	-1.358181108	1.74E-05	0.000100113
IGKV1OR22-1	8.63662725	3.844718374	-1.167590101	1.85E-05	0.000105214
F2RL2	1.667919899	4.360284495	1.386372264	0.000116412	0.000501006
SLC7A11	7.313224271	23.9295582	1.710214251	1.76E-17	7.23E-15
CIBAR2	9.410867174	3.256162363	-1.531155029	1.83E-06	1.50E-05
TM4SF20	0.079803929	7.964777254	6.641030428	0.000734744	0.002382604
AL138760.1	1.17480545	3.825043033	1.703054115	2.62E-09	5.91E-08
LINC02577	1.943170025	4.113489481	1.081950613	1.36E-10	4.91E-09
AL365181.2	1.43446616	9.98020724	2.79855584	5.73E-27	4.94E-23
DNER	3.869696895	10.03523695	1.37478221	0.002846616	0.007457029
MACROD2	9.592690241	3.874201844	-1.308036352	0.000596039	0.002003593
TAS1R1	1.857998669	0.710162842	-1.387527688	2.87E-06	2.19E-05
NNAT	22.78373276	4.160687432	-2.453110313	0.001758615	0.004954322

KIF19	3.620276426	1.101334426	-1.716847241	2.67E-08	4.30E-07
TEPP	3.28931616	1.34177944	-1.29364014	2.42E-12	1.71E-10
KLK5	3.939814575	10.60878518	1.429059824	0.000260292	0.000992733
RHCG	1.641808175	5.952795355	1.858281723	0.000744211	0.002408324
PTPRT	1.500941952	0.553723975	-1.438629288	0.000127773	0.000542864
LINC00460	1.09439474	4.51502623	2.044601167	1.80E-11	9.02E-10
NFE4	1.724089163	11.23815546	2.704498971	5.99E-09	1.20E-07
SLCO4A1	5.025463878	12.78395676	1.347005758	1.79E-12	1.31E-10
CDA	39.3069993	103.4662587	1.39630223	1.06E-09	2.78E-08
SCGB3A2	1542.871251	763.7513517	-1.014442744	1.47E-05	8.67E-05
PTGR1	36.5759353	84.94042705	1.215556605	1.19E-14	1.82E-12
F5	5.169594423	11.37114303	1.137254277	0.004127447	0.010181745
AL096854.1	1.020828264	2.139473702	1.067515767	0.012438891	0.025687494
COL7A1	5.882186565	11.9249929	1.019563959	1.63E-07	1.98E-06
OPN3	3.798089797	10.37805731	1.450190487	6.28E-19	3.87E-16
AC005077.4	2.325195247	6.404328005	1.461695334	3.94E-16	1.03E-13
NMRAL2P	3.423549873	12.56314173	1.875632358	1.53E-12	1.16E-10
CPB2	4.94977237	1.71994638	-1.524998591	0.010722139	0.022748865
APOBEC4	1.833606337	0.663250273	-1.467058665	0.00162805	0.004651803
VSIG10L	5.293529278	14.08338743	1.411692567	1.93E-08	3.25E-07
HLF	14.97913264	6.790298907	-1.141407099	6.28E-14	7.48E-12
FAM83B	1.080917934	2.436715096	1.172680585	1.79E-05	0.000102266
ANXA10	11.74268143	27.16575615	1.21002732	7.96E-07	7.52E-06
IGLV6-57	819.3519553	391.6099705	-1.065065803	7.78E-06	5.13E-05
C1orf194	10.88987034	3.71654918	-1.550951173	2.30E-05	0.000126626
FAM216B	9.480281622	3.373775068	-1.490566125	0.000131367	0.000555917
HHIPL2	4.568342966	15.05785738	1.72077363	4.56E-17	1.64E-14
AL357093.2	2.086412864	0.88850806	-1.231567901	0.00049829	0.001717598
IGHV3OR16-6	5.171669645	2.409797063	-1.101718465	1.63E-05	9.47E-05
CBR1	120.3626674	297.2863958	1.304465461	0.000148904	0.00061863
AL513548.4	3.414885361	0.852046243	-2.00283351	2.67E-09	5.98E-08
CCDC114	5.26795526	2.588684904	-1.025023722	5.23E-05	0.000254127
NQO1	185.5598432	430.4237196	1.213873052	1.69E-12	1.24E-10
FCER2	2.30387839	0.888898839	-1.373973419	1.04E-07	1.36E-06
COPDA1	2.318234854	1.113598566	-1.057797471	5.84E-10	1.67E-08

TNS4	13.38727142	40.0145569	1.579662992	4.59E-09	9.50E-08
CTNND2	6.453972433	2.444390369	-1.400712714	1.56E-06	1.32E-05
DPYD-AS1	3.586682319	1.212811544	-1.564294578	0.000199672	0.000793082
AHNAK2	11.43029075	28.26133327	1.305967427	1.16E-19	9.07E-17
TRIM16L	7.557636629	19.34080799	1.355641006	1.41E-14	2.08E-12
LRRC55	1.872742269	0.697993101	-1.423867684	0.025129456	0.046619506
APOBEC1	1.913009252	4.414073634	1.206266845	5.72E-07	5.69E-06
CYP4F3	4.27094962	11.99575205	1.489894822	1.44E-10	5.13E-09
AC073648.6	6.784652091	3.34220321	-1.021475384	0.000685117	0.002244873
C8orf34-AS1	6.979150444	3.158338115	-1.143885804	8.27E-09	1.58E-07
IGFBP1	5.304721863	10.65426537	1.006082105	1.62E-11	8.27E-10
KYNU	3.267068948	13.69776933	2.067872164	4.30E-14	5.30E-12
DNAI1	3.854449049	1.396369331	-1.464844082	0.006144364	0.014220121
FAM83A	46.34925488	111.3647366	1.264674428	4.20E-12	2.74E-10
GPAT3	6.596923321	13.74140594	1.058664379	3.81E-12	2.55E-10
TMEM213	5.84433815	2.69614235	-1.116142985	0.000434231	0.001525952
DRC7	1.618978897	0.702282036	-1.204961744	0.010302274	0.021980849
PACRG	2.449241381	1.11107623	-1.140377162	0.001169235	0.003515361
ERICH3	2.671098669	0.999460178	-1.418212279	0.010895821	0.023061084
TRIM31	4.995305133	13.93366223	1.479929784	5.73E-07	5.69E-06
PIGR	976.4467013	457.699116	-1.093141792	9.63E-10	2.56E-08
SPAG8	3.359946895	1.522915984	-1.141602078	8.21E-11	3.21E-09
TOGARAM2	1.73076185	0.77278832	-1.163262032	0.004540877	0.011022036
CFAP161	1.549996324	0.646668716	-1.261166071	0.00330935	0.008456139
AKR1C3	89.22027852	200.7210048	1.169748041	1.19E-07	1.53E-06
UGDH	84.67433365	214.8608514	1.343406008	2.48E-20	2.52E-17
AP003119.3	1.597816286	3.30491612	1.048512116	8.83E-17	2.99E-14
TINAG	1.322004119	2.78419153	1.074531788	9.01E-07	8.32E-06
DNAAF6	1.683669265	0.587611202	-1.518674963	0.001293817	0.003831278
FAIM2	4.830294613	1.564793101	-1.626139271	1.78E-09	4.32E-08
LRRTM1	1.606997085	0.750334358	-1.098761786	0.000767142	0.002464507
LINC01214	0.753303866	3.126287227	2.053146494	0.018571603	0.036089261
CDC20B	2.806656907	1.207797951	-1.216473586	9.69E-05	0.000430071
IGHJ5	55.63483447	27.3190651	-1.026080363	3.04E-10	9.58E-09
LINC00942	9.738826236	31.25852329	1.682429816	2.29E-06	1.81E-05

AC207130.1	1.113954626	2.381098497	1.095936831	0.011851869	0.024671182
AL133304.2	2.538598479	1.139659358	-1.155429557	0.000219296	0.000858772
TMEM229A	3.680910266	0.658660178	-2.482456347	8.80E-08	1.18E-06
S100P	873.0769139	2556.864502	1.55019505	1.01E-08	1.88E-07
MYCN	11.26946109	3.517134973	-1.679945921	0.002136335	0.005841076
TGFBI	94.20016901	199.2823568	1.081012436	2.26E-06	1.79E-05
DKK1	12.45304975	37.55203839	1.592392121	1.36E-07	1.70E-06
LINC02015	0.557347529	1.538357104	1.464741348	1.10E-10	4.14E-09
CDHR2	1.103542142	5.035543511	2.19000578	1.99E-05	0.000111814
ALDH3A1	28.62779981	86.46177944	1.594645732	0.011386254	0.023870745
LAMA3	14.79205437	33.83124249	1.19353373	2.16E-08	3.60E-07
VEGFC	7.073397782	19.25444617	1.444716326	1.49E-11	7.70E-10
MAGEA1	4.207617997	9.940272063	1.240281608	0.000717018	0.002334772
C9orf24	14.15331343	4.760770902	-1.571872732	0.000721612	0.002347075
VGF	5.31120564	10.83997548	1.029250199	8.34E-05	0.000378024
IGHA2	978.0075221	485.0762889	-1.011633901	2.38E-05	0.000129826
ADGRF4	3.807624968	9.342942077	1.294985536	8.68E-18	4.05E-15
UCN3	51.95079138	10.92246612	-2.249847096	0.008687319	0.019017092
TUBA4B	5.849459886	2.04127985	-1.518829436	0.003669085	0.009224658
SPAG6	8.637466413	3.814386817	-1.179157041	0.001456768	0.004238493
TMPRSS11E	10.40695583	36.08197889	1.793730341	1.29E-16	3.85E-14
VWA3A	3.340546831	1.113288388	-1.585256925	8.70E-05	0.000392014
IFITM5	1.808130545	0.654045355	-1.467036254	0.017387647	0.034095673
GLB1L3	12.04288859	5.61520403	-1.100771127	1.42E-06	1.21E-05
KRT6A	63.95055076	169.1438622	1.403222137	8.18E-12	4.82E-10
MT1A	7.382053232	27.09474706	1.875919133	0.001685588	0.004787768
MELTF	5.978807985	15.53913074	1.377976018	5.36E-09	1.09E-07
ABBA01000935.2	3.326910963	0.797749249	-2.060176007	7.64E-07	7.27E-06
LILRA2	8.702599176	2.054431284	-2.082707274	2.13E-06	1.71E-05
MARCHF4	0.382307795	2.054127186	2.425718992	0.000860018	0.002714374
MGAT5B	0.686564512	1.752613388	1.352040592	0.000468182	0.001627374
AC092128.1	1.648443283	0.536656489	-1.619033422	0.000955685	0.002955244
LHFPL3	2.649429721	0.928948702	-1.512011023	1.04E-14	1.64E-12
AC007906.2	19.71751888	7.45392944	-1.403404957	0.001798969	0.005052337
FOSL1	11.59083105	38.26098354	1.72288995	9.12E-23	1.97E-19

WDR86-AS1	26.50747947	12.79671393	-1.050626106	3.57E-07	3.86E-06
UCA1	1.171965653	3.748499249	1.677382824	1.52E-06	1.29E-05
FAM83A-AS1	4.116926236	8.535453825	1.051900263	9.60E-09	1.79E-07
GSEC	2.664042522	5.607425273	1.073721379	5.77E-19	3.69E-16
LRRC71	2.613023764	0.896914822	-1.542677357	0.010899051	0.023065091
IGHV2-70D	77.18891825	36.26438128	-1.089840505	0.001436989	0.00418534
AC010378.1	2.993395944	1.342065369	-1.157328177	1.25E-07	1.59E-06
GCLM	22.86836933	48.38258422	1.081134334	1.52E-17	6.58E-15
ARNTL2	9.96166616	24.29122152	1.285976071	4.00E-15	7.42E-13
AL691432.4	2.087973891	1.03867903	-1.007353767	9.29E-08	1.24E-06
AL365181.3	5.241702788	22.54241325	2.104534511	3.70E-26	2.13E-22
MUC2	0.26857199	2.091241872	2.960979178	0.006698943	0.015295961
PCP4	71.06684994	32.48122821	-1.129570567	5.06E-07	5.14E-06
AKR1C2	83.91283897	279.8396469	1.737636903	3.44E-09	7.39E-08
IL36RN	2.027807098	4.81894638	1.248797331	0.000652173	0.0021561
CRYM	35.8752749	17.25532705	-1.055948069	1.33E-10	4.82E-09
TFF2	104.3928479	255.3303132	1.290341952	0.023093185	0.043343029
RN7SL8P	2.577384854	1.025831079	-1.32911479	8.66E-07	8.04E-06
AL590666.2	3.284463181	10.57656387	1.6871415	3.08E-24	1.06E-20
ANKRD44-AS1	6.301206464	2.29325526	-1.458231133	1.87E-06	1.53E-05
FOXI3	5.292256084	2.131741393	-1.311850441	0.000104593	0.000457155
KCP	0.898743029	2.152050615	1.259731429	1.17E-11	6.42E-10
DCBLD2	26.33596603	56.52695458	1.101904593	0.000260688	0.000993805
LRRC18	1.73969924	0.691356489	-1.331336199	1.20E-05	7.30E-05
UCHL1	64.9235552	163.0800988	1.328766827	1.08E-09	2.83E-08
GFRA3	58.38973207	23.32505437	-1.323834247	1.43E-11	7.45E-10
IGKV6D-41	2.995553169	0.441821585	-2.761286634	3.09E-06	2.34E-05
HAGLR	42.14359449	21.06196428	-1.000673378	6.57E-11	2.67E-09
CFAP77	3.515925856	1.313230055	-1.420784974	0.00088629	0.0027795
AC244258.1	6.319132066	0.781687568	-3.015062419	0.012792172	0.026313146
NTSR1	0.30695076	4.17774638	3.766645764	3.42E-06	2.56E-05
ZBBX	2.361917871	0.916524727	-1.365713091	0.003264381	0.008356427
MLXP1	1.225975539	4.333699454	1.821668911	1.83E-06	1.50E-05
SERPINB5	7.640573447	23.02901113	1.591699637	4.08E-12	2.67E-10
RSPH4A	7.393605133	3.409496585	-1.116719256	0.00111575	0.003375811

IGLVI-70	5.510802155	2.657166872	-1.052373501	0.000129732	0.000550104
IGKV2D-30	12.03487079	5.762789959	-1.062381309	7.66E-08	1.05E-06
PPP2R2C	1.806146388	7.664611544	2.085297848	3.08E-15	5.97E-13
C20orf85	50.91335824	16.81170669	-1.598578037	0.000313592	0.001155662
IGLV1-36	152.3475382	46.70351236	-1.70576323	2.68E-05	0.000143768
HEPACAM2	9.044188086	3.952762842	-1.194129596	0.000381061	0.001366089
SNORA80D	3.378473257	1.446384631	-1.223920182	0.025439039	0.047095787
ZMYND10	12.23528302	5.392050205	-1.182141639	0.003017644	0.007818422
CCDC60	1.706203739	0.724455738	-1.235820477	8.89E-05	0.000399303
IGHD3-22	4.28971711	2.108632309	-1.024574963	3.32E-07	3.62E-06
GJB2	35.18754043	71.14345717	1.015666408	8.03E-07	7.55E-06
CFAP52	4.41387161	1.591985109	-1.471217824	0.000112996	0.000488013
AKR1C4	1.220445184	4.200169126	1.783039923	4.02E-06	2.95E-05
IGHV3-20	92.62892687	45.45198982	-1.027119345	0.000232919	0.00090473
INSL4	2.553595691	15.56922876	2.608095453	7.07E-09	1.39E-07
MIR9-1HG	0.422403802	1.69600888	2.005448997	9.76E-07	8.91E-06
LRRC66	0.773837643	1.882417418	1.282483761	9.18E-10	2.44E-08
LINC02323	0.661469772	2.17695041	1.71856141	4.63E-13	4.27E-11
LHFPL3-AS2	12.30941838	4.207791462	-1.548627484	1.49E-11	7.68E-10
HTR3A	6.591991698	16.30738613	1.306739224	8.20E-08	1.11E-06
SCGB2A1	28.78323612	11.83744501	-1.28187108	0.000215347	0.000845034
TTC29	1.861749873	0.72104597	-1.368496113	0.007183407	0.016211749
BPIFB2	66.92536952	23.8654873	-1.487627405	0.005701868	0.013369951
IGHD6-25	7.946404246	3.584844467	-1.14839166	0.00029133	0.001092043
AQP5	195.7164171	66.8560806	-1.549634092	3.32E-12	2.27E-10
GGTLC1	63.91092744	27.7869362	-1.201655852	8.94E-12	5.20E-10
IGKV2-40	2.039293156	0.902031421	-1.17681959	0.000152631	0.00063153
PRDM16-DT	2.917673067	1.136286612	-1.360491455	7.64E-09	1.48E-07
FGA	155.8575333	521.9450536	1.743670051	0.002419101	0.006489796
F11	1.650482193	0.592185792	-1.47876579	1.39E-06	1.20E-05
SFTPC	1536.27648	548.9765891	-1.484621345	3.00E-05	0.000158525
GPX2	120.9070326	388.8273179	1.68523142	5.07E-05	0.000248163
RN7SKP97	1.567554183	3.795030191	1.275596052	1.20E-07	1.54E-06
SERPINA5	2.03435583	4.954846038	1.284268186	0.005796926	0.013560012
IRX1	4.654612104	1.428854645	-1.703801784	1.03E-06	9.29E-06

STOML3	4.309020786	1.60782418	-1.422250405	0.001942354	0.005384924
SNTN	5.935856147	2.436967077	-1.284369369	9.70E-05	0.000430407
PSMD10P2	0.666524588	1.95193627	1.550175948	1.05E-16	3.36E-14
HOXD1	5.256912294	2.224458333	-1.24076159	1.39E-09	3.51E-08
SDCBP2	15.6335526	37.19926469	1.250628448	2.27E-13	2.33E-11
IL11	1.463487706	3.230446721	1.142323055	2.75E-07	3.09E-06
KLK12	57.07184512	24.32274515	-1.23047314	0.000163682	0.000669523
TCTE1	1.583579848	0.546854918	-1.533959575	0.000887851	0.002782878
AP003119.1	1.070224778	2.64087903	1.303104382	4.25E-13	3.99E-11
IGKV1D-16	77.02253219	21.78826421	-1.821729287	1.96E-06	1.59E-05
TEKT1	6.357652218	2.173867418	-1.548230144	0.002188092	0.005958985
LINC02057	0.735271039	1.480337978	1.009578531	3.59E-07	3.87E-06
AC245041.2	1.681366793	3.410180806	1.020213747	2.70E-07	3.04E-06
DUSP13	1.852884094	5.371687227	1.535602667	6.20E-13	5.30E-11
HMGA2	2.04259493	6.861564139	1.748134356	0.010808626	0.022901814
FCRLA	5.913581369	2.885472131	-1.03522472	2.84E-07	3.17E-06
LINC02331	0.762352788	1.782877322	1.225676754	0.000236071	0.000914711
AKAP14	2.955801901	1.092484836	-1.435936328	0.0009074	0.002830276
NWD1	3.183891065	1.318708197	-1.271665615	0.009494472	0.020471713
AC018629.1	1.761058999	5.010450956	1.508497213	3.70E-06	2.75E-05
SERPINA4	3.155930418	8.38162377	1.409164367	0.004011961	0.009939481
GUCY2EP	0.447783333	1.624262568	1.858912132	7.34E-09	1.43E-07
PCDH7	4.644557605	10.57083689	1.186476507	5.84E-08	8.32E-07
AC245041.1	1.561354626	4.660426639	1.577663783	5.75E-06	3.96E-05
CA10	2.955623321	1.468957377	-1.008669881	8.93E-06	5.73E-05
BHMT2	10.9850249	27.44427691	1.320967188	0.001388197	0.004064037
SPEF1	4.968320406	2.287927186	-1.118717077	0.002240597	0.006080882
UGT2B7	1.231432319	2.534566325	1.041401579	7.42E-05	0.000342983
GPC6	6.333258112	13.41061469	1.082355583	1.98E-05	0.000111202
BCL2L10	1.335188276	2.917354508	1.127617517	0.000185867	0.000745973
CPS1	48.61801179	103.6115755	1.091622389	0.000628866	0.00209355
DCDC2B	1.936212484	0.873095423	-1.149026041	0.000209936	0.000826477
BCAN	0.697589354	2.495146858	1.838674804	4.33E-25	1.87E-21
SUSD2	177.2152301	75.37204686	-1.233401119	2.07E-07	2.43E-06
RN7SL3	41.34632662	88.19646072	1.092961602	0.000198917	0.000791175

ASPH	48.28669278	99.74620663	1.046636322	2.26E-12	1.61E-10
KCNF1	1.105065526	3.124664071	1.499569177	2.70E-07	3.04E-06
BTBD11	1.447314956	2.898156557	1.001756625	6.59E-05	0.000308724
AC026785.3	0.884328644	6.181752117	2.80536128	1.41E-12	1.08E-10
PRR20G	1.060960203	2.514834426	1.245092877	5.90E-06	4.05E-05
ACTL8	0.528036755	2.674657923	2.340644128	1.35E-07	1.69E-06
HHIP	5.641936312	2.775978552	-1.023193959	0.002877808	0.007522784
APCDD1L	0.989914195	2.977850751	1.588896064	1.80E-06	1.49E-05
CCDC190	1.798368885	0.624658538	-1.5255493	0.002153145	0.005878654
CFAP65	1.68439512	0.635819331	-1.405541817	1.98E-05	0.000111308
AKAP12	11.10699702	41.29569898	1.894522719	1.45E-07	1.79E-06
NEIL3	3.213553549	8.660948087	1.430355448	3.77E-19	2.52E-16
AP000695.1	1.469155957	3.001160178	1.030532768	8.10E-12	4.80E-10
LINC01765	2.922737516	1.053067145	-1.472722843	0.003644999	0.009180589
AL033397.2	0.825799366	2.400449863	1.539441587	1.19E-11	6.46E-10
EREG	13.21527148	28.04037705	1.085299686	1.22E-05	7.38E-05
EFCAB1	3.834182256	1.423537978	-1.429437934	0.011898318	0.024740006
ELF5	10.89739385	5.337480328	-1.029752398	1.75E-06	1.45E-05
IL5RA	1.470807414	0.627990847	-1.227792917	4.90E-06	3.47E-05
AC002511.2	1.559748352	0.622660246	-1.324796208	0.003678524	0.009243443
IGKV1OR2-6	17.13837636	7.444958128	-1.202894799	2.75E-06	2.11E-05
UMODL1	4.914119392	1.943084221	-1.338584475	4.73E-08	6.99E-07
MEGF11	2.10608251	0.671314003	-1.649502317	2.04E-09	4.86E-08
PTPRN	0.900601014	3.625172473	2.009089628	2.23E-06	1.77E-05
AP000695.2	0.946955196	2.034259085	1.10313536	5.47E-14	6.69E-12
AKR1B15	1.465508492	6.579270423	2.166526284	9.79E-19	5.63E-16
AL033397.1	2.324075792	7.766321107	1.74057424	2.67E-12	1.87E-10
NR0B1	4.662004943	14.78128033	1.664748799	4.43E-10	1.31E-08
DLGAP1-AS2	3.368688593	6.965000342	1.047936354	1.38E-11	7.22E-10
KRT86	2.740231622	5.57097056	1.023630848	7.22E-06	4.82E-05
CELF3	2.63998834	0.970185724	-1.444198701	7.72E-05	0.000354669
GFRA1	2.724857098	1.225185314	-1.153180592	1.66E-09	4.07E-08
SRGAP3-AS2	7.959018758	2.661366803	-1.580423213	0.00135815	0.003993008
SBK3	0.588187072	1.500826298	1.351410032	1.14E-11	6.31E-10
TFF1	201.6953874	512.8443865	1.346343041	0.022380462	0.042138556

BNC1	0.616802345	1.709638593	1.470811226	0.009828491	0.021089158
IGHJ3P	75.04561895	34.92126223	-1.103662146	2.10E-10	7.08E-09
FLNC	5.290193219	17.20118395	1.701115547	2.37E-11	1.13E-09
NAMPT	63.8612903	139.7781345	1.13012509	2.48E-18	1.26E-15
EPGN	0.481695817	1.898641257	1.978773036	0.003820982	0.009537534
AMPD1	1.582925856	0.744455055	-1.088337025	3.04E-11	1.39E-09
LINC01269	1.451325475	4.33404515	1.578343087	2.84E-20	2.67E-17
NTRK2	2.028582446	0.71612821	-1.502182133	0.010912731	0.023085608
LASTR	0.728230038	1.544565164	1.084734583	1.10E-11	6.12E-10
SAXO2	4.369809696	1.870916735	-1.223825099	3.95E-05	0.000200538
CRYBG2	2.456360456	5.975510929	1.282539791	2.39E-17	8.96E-15
MS4A1	13.85549677	5.689107514	-1.284184187	1.14E-07	1.47E-06
NELL1	8.614860203	3.776311885	-1.189849464	0.000100337	0.000442302
ATP8A2	2.197016223	0.988070902	-1.152859048	4.25E-09	8.89E-08
IL22RA1	3.312402471	7.296521995	1.139330967	4.13E-07	4.34E-06
RNU6-247P	4.950219518	1.713147131	-1.530843443	5.14E-05	0.000250944
AKR1B10	70.61239601	253.0757094	1.841575666	3.65E-14	4.57E-12
CCDC78	7.441889227	3.651175956	-1.02730772	0.000118302	0.000508508
AL445493.3	2.739215843	1.158266598	-1.241795594	1.46E-09	3.63E-08
IL20RB	6.902512294	18.36534337	1.411792413	7.41E-11	2.94E-09
CTSV	4.5902673	10.4214028	1.182899416	2.81E-10	8.98E-09
COL11A1	18.66847909	37.34170977	1.000183589	8.82E-05	0.000396675
KRT81	14.56921229	74.51041434	2.354519208	1.17E-06	1.04E-05
PTPRR	1.022281686	2.868998156	1.488754261	8.53E-06	5.51E-05
LYPD3	12.51071426	42.63690772	1.768938651	5.07E-15	8.93E-13
AC089983.1	0.840136819	4.586112363	2.448575503	9.16E-17	3.04E-14
NCCRP1	9.564107858	19.32516626	1.014778521	0.00192322	0.005337887
C8orf34	1.36936185	0.651142213	-1.07245915	2.22E-08	3.68E-07
STC1	19.28172795	40.30841154	1.063846586	1.33E-09	3.37E-08
HNF4A	1.857102218	4.336358675	1.223430866	0.004561245	0.01106522
LINC01983	1.89728403	3.901778962	1.040196379	0.003131498	0.00806808
OSGIN1	10.09714455	21.71789399	1.10493685	2.13E-14	2.92E-12
SPOCK1	5.880943663	12.74707302	1.116046438	4.71E-05	0.000232793
CHAD	8.708161027	3.688556899	-1.239311595	0.000111741	0.00048295
INHA	10.79920336	38.79762213	1.845043343	2.78E-06	2.13E-05

GRAMD1B	1.834414512	5.673520697	1.628924603	0.000247755	0.00095186
AC002563.1	2.766001711	1.006269809	-1.458784866	2.94E-07	3.26E-06
POPDC3	1.393572433	5.145837432	1.884617889	5.18E-09	1.06E-07
DNAH12	1.401606401	0.576494809	-1.281701746	0.001758612	0.004954322
SCGB3A1	2626.599474	738.441656	-1.830640379	2.71E-07	3.05E-06
HAS3	41.87868105	20.5093959	-1.029931008	9.59E-06	6.09E-05
IL1R2	3.711650253	9.36087821	1.334583113	8.71E-08	1.17E-06
VNN1	4.357130292	11.48075526	1.397767393	0.008294893	0.018294347
C11orf16	4.833365463	1.744462158	-1.470245782	7.27E-08	1.00E-06
STMND1	2.959596768	1.417281148	-1.062274653	3.18E-07	3.48E-06
FBN2	1.023931305	3.545419945	1.791837591	0.01488939	0.029934945
HOATZ	2.328788213	0.849396175	-1.455069926	0.013283863	0.027162666
HHATL	2.208639544	0.588916598	-1.907022745	0.000783417	0.002511184
TXNRD1	103.0781652	336.5190538	1.70694943	5.84E-18	2.80E-15
TEKT2	9.469486755	4.083971995	-1.213313261	4.36E-07	4.54E-06
LOXL2	20.66484607	42.53766059	1.041562075	2.28E-10	7.60E-09
KIAA0319	2.113104246	5.847341393	1.468416883	2.41E-10	7.91E-09
AC021218.1	0.640289544	1.533311134	1.259854118	0.00670977	0.015317091
TTLL10	1.870014385	0.682443716	-1.454267398	4.67E-06	3.32E-05
AC068587.2	4.654216413	2.237848566	-1.056425882	4.57E-06	3.27E-05
PGC	3379.53121	1633.009474	-1.049289977	4.28E-05	0.000215023
IGHV1-14	7.578620722	3.019955191	-1.327408164	4.82E-06	3.42E-05
RASGRF1	7.565896261	3.431197473	-1.140798837	2.15E-05	0.000119251
LY6G6C	0.65386071	1.918897951	1.553222749	0.0039772	0.00986044
CCDC17	8.806911914	3.7339125	-1.237948116	0.000735277	0.002383883
ATP13A4-AS1	7.802629278	2.948005669	-1.404221057	8.11E-05	0.000369378
AL391427.1	3.509952155	9.508612022	1.437783401	8.22E-06	5.34E-05
IGF2BP1	1.788237833	5.835810656	1.70639445	0.000171976	0.00069851
CAPSL	10.0315775	3.498721721	-1.519648667	0.001861176	0.005198282
LINC01843	2.37101033	5.129526366	1.113323673	2.19E-06	1.75E-05
NEUROD1	7.248514132	1.405178074	-2.366932318	0.000166036	0.00067757
TSKU	43.99974937	88.48722234	1.007973837	3.36E-27	4.94E-23
SLCO4A1-AS1	1.087925412	2.392368648	1.136860067	9.20E-07	8.47E-06
AC008268.1	13.7400225	5.918815164	-1.215004057	9.96E-08	1.32E-06
AC009549.1	0.978416477	2.243723361	1.197374206	0.000238804	0.000923654

CALB2	3.024123764	14.58563429	2.269959037	0.00010331	0.000452403
TRIM15	1.513982953	3.11830929	1.042415068	5.08E-07	5.15E-06
SCGB1A1	388.866539	125.8285958	-1.627815274	0.000901559	0.002816638
IGKV2D-40	59.79872452	28.62585178	-1.062796091	0.000159798	0.000656545
SLC13A2	9.102895817	2.943141598	-1.628968615	3.99E-12	2.63E-10
AC106045.1	1.967324588	6.816029577	1.792696589	8.77E-12	5.12E-10
BMX	3.242266667	1.496608128	-1.115306237	0.002475634	0.006622926
IGKV3D-7	4.312938593	1.858555464	-1.214489432	1.29E-06	1.12E-05
CNGA3	7.113729341	3.333012978	-1.09377915	0.000826634	0.0026234
SLC47A1	10.75559753	5.317678005	-1.016219349	6.63E-13	5.64E-11
CCDC151	3.63182763	1.737693033	-1.063522483	3.76E-06	2.79E-05
KLK13	16.53411445	5.786760792	-1.514617865	1.68E-05	9.72E-05
AL109761.1	0.898957921	2.31580929	1.365190958	0.001319418	0.003893727
GJB4	0.920673131	1.940257172	1.07548694	7.40E-06	4.92E-05
AC234772.1	0.708371166	1.741232309	1.297531301	0.007222168	0.016286446
IGKV6D-21	27.51758371	11.63177766	-1.242282196	2.91E-08	4.64E-07
ANXA13	0.933440494	2.619131694	1.488458642	0.00256046	0.006818149
C6	3.641079278	1.487265027	-1.291704398	1.46E-05	8.64E-05
MYT1	1.790521229	0.57961571	-1.62721102	0.014480548	0.029238949
BEND6	1.040406274	2.329021448	1.162576921	5.03E-13	4.56E-11
ANO3	0.609492839	1.820653279	1.578775029	0.010384001	0.022127063
AKR1C1	51.31136375	175.7387433	1.776082007	6.98E-07	6.73E-06
DNAAF1	3.932873828	1.436782719	-1.452742	0.000263474	0.00100221
SLC46A2	9.180098416	4.287734631	-1.098294	4.23E-06	3.07E-05
LINC01842	1.526228771	3.218905328	1.076598918	6.20E-06	4.23E-05
RAB3B	1.500843093	3.723624932	1.310934606	6.99E-06	4.69E-05
IL1A	1.601643156	5.880187022	1.876309288	0.000251897	0.000966268
EPS8L3	4.093120596	9.769856831	1.255136247	0.001990954	0.005501103
GP2	8.493130798	2.785710109	-1.608251334	4.55E-05	0.000225974
C9orf135	10.18032497	4.887931489	-1.058487646	4.84E-05	0.000238098
PTPRZ1	4.888441381	2.09959276	-1.219265026	1.55E-07	1.90E-06
ALB	177.8481557	3.668350342	-5.599370775	0.001485039	0.004309534
NKAIN1	0.772108238	1.760174454	1.188843412	1.08E-06	9.72E-06
CD109	8.894693663	19.58768839	1.138930326	9.22E-15	1.49E-12
ADAMTS7P3	1.47276166	0.728930123	-1.014671549	0.000301952	0.001123869

CNGA4	2.043770152	0.876062022	-1.22212804	0.008059657	0.017873755
HTR1D	1.882583777	6.586621858	1.806824659	1.05E-15	2.47E-13
MAGEA6	10.00112471	21.12819276	1.079007116	0.015854249	0.031536725
LINC00973	2.650525412	9.013594672	1.765824203	0.004294919	0.010515209
STC2	7.512817364	15.20494795	1.017114942	1.21E-11	6.52E-10
KLK14	43.52615982	15.31656257	-1.506790186	8.92E-05	0.000400274
MIR4539	13.76100627	6.511918033	-1.079431526	1.02E-07	1.35E-06
C1orf87	1.952674905	0.667259699	-1.549131502	0.008287107	0.018279511
GJB3	8.575299303	28.81905574	1.748764135	1.90E-17	7.62E-15
CABCOCO1	5.726029531	2.782452117	-1.04117825	9.90E-05	0.000437584
BPIFB1	282.7889856	125.3315612	-1.173976167	0.001493935	0.004328804
IGHV3-73	318.2394046	140.9215596	-1.175220136	8.07E-06	5.27E-05
AC104971.3	1.383275095	0.664261339	-1.058265243	1.85E-09	4.48E-08
KRT83	0.686284728	2.041453142	1.572717299	0.000346742	0.001261929
RGS20	1.284188213	3.010529372	1.229160531	6.75E-12	4.10E-10
TSPAN19	2.17143365	0.726706079	-1.579203991	0.00199666	0.005511574
PRSS3	3.711102535	8.495486202	1.194848654	0.00557892	0.01314237
BANCR	6.431407224	2.094193716	-1.618739541	0.001322409	0.003901887
GABRA3	1.617797465	3.31426612	1.034658443	0.003588307	0.009069567
SNCG	47.25884892	103.533819	1.131445701	1.26E-10	4.66E-09
GREB1L	1.011919455	2.41163873	1.252919342	7.21E-11	2.89E-09
ANXA8	1.258674018	2.884615779	1.19647448	4.40E-06	3.17E-05
ANLN	18.11289753	37.89978074	1.065172149	1.93E-15	4.13E-13
AC090001.1	1.516118885	0.714405738	-1.085567313	0.003482096	0.008836054
CXCL5	13.8689192	31.55226257	1.185888099	0.010511738	0.022360603
IGLV1-41	31.71726381	12.7419931	-1.315677357	1.41E-05	8.39E-05
AZU1	2.405127376	1.045523019	-1.201888476	2.02E-05	0.00011302
CASC15	0.655397655	1.477603484	1.172816756	5.66E-06	3.91E-05
MIR31HG	0.643901267	2.202178552	1.774020052	2.65E-10	8.55E-09
SH3PXD2A-AS1	1.020139544	2.169428893	1.08854879	5.39E-09	1.09E-07
CR2	11.62645215	5.44933832	-1.093257954	1.01E-06	9.19E-06
KLK8	0.989665906	2.900442213	1.551259392	7.49E-09	1.46E-07
TCTEX1D1	2.026812801	0.897331967	-1.175499133	0.000289492	0.001087515
C4BPA	488.0824581	186.1957019	-1.390305131	3.57E-12	2.40E-10
KCNK17	5.35153872	2.575347268	-1.055186784	1.98E-05	0.000111159

C1QL2	6.841750824	3.188844126	-1.101331983	0.002200364	0.005985796
AC005336.1	1.428124651	6.30179679	2.141641325	1.27E-07	1.61E-06
CBR3	6.272463561	13.08116742	1.060387209	3.08E-07	3.39E-06
UGT1A6	0.95063251	3.754009358	1.981472602	0.000332995	0.001218581
FDCSP	40.81953295	16.82100061	-1.278996147	6.10E-06	4.17E-05
CYP24A1	42.22430323	134.8868682	1.675604381	1.42E-08	2.53E-07
NRK	2.376725475	5.460113934	1.199955782	0.021444629	0.040651517
MSTN	7.402437959	0.805857036	-3.199404671	0.002037575	0.005603007
IGHD2-2	5.402916603	2.673382719	-1.015072026	1.04E-06	9.42E-06
LINC01559	1.085447275	3.661584631	1.754178492	0.00063047	0.002097676
KRTDAP	3.064655133	0.409654235	-2.903246101	9.52E-05	0.000423404
SLC25A47P1	1.583832953	0.754793648	-1.069265996	0.007408921	0.016635851
UPK1B	6.709028707	30.97598484	2.20697433	0.004997539	0.011977159
MAGEC1	1.025247972	3.512535383	1.776539868	0.000713686	0.002326559
PKIB	9.132298352	21.3791332	1.227153463	1.48E-07	1.83E-06
FCRL1	2.128142522	0.881762363	-1.271132967	2.84E-09	6.29E-08
AC239859.5	0.665856401	2.021695697	1.602282878	0.000303549	0.001128534
MIR3189	26.16036343	11.93946701	-1.131644149	2.67E-07	3.02E-06
DLEC1	3.361416096	1.541862978	-1.124394577	0.00010738	0.0004675
PLAU	110.855931	221.7962611	1.000549087	1.24E-06	1.09E-05
AC138649.5	2.048311977	0.854717964	-1.260915118	2.79E-08	4.47E-07
AL645924.1	4.057171356	1.630840027	-1.314858965	0.001330986	0.003925179
FHOD3	2.319154753	4.68375485	1.014066475	0.000153332	0.000633823
FAM177B	2.872004246	6.696011885	1.221244207	0.000606121	0.002030764
FAM183A	17.15545475	8.043938525	-1.092693408	0.001021743	0.003133229
C22orf15	2.391020722	0.834642964	-1.518395541	0.00073283	0.002376904
CIDEC	0.500359189	2.293951161	2.196798648	1.06E-05	6.63E-05
RPSAP52	0.552137516	1.507459153	1.449019375	0.001659742	0.004725925
IGKV2-29	66.99589011	23.91896257	-1.485917778	0.005507576	0.012994305
RPL26P30	7.726024271	2.852375068	-1.437562511	1.53E-05	8.98E-05
DNAI2	3.422248226	1.089788388	-1.650896383	1.75E-05	0.00010058
SLC2A1	52.81480684	140.8923958	1.415579393	4.03E-21	5.36E-18
IGHV3-22	3.739635741	1.528947609	-1.290358779	2.92E-05	0.000154847
AL355916.1	1.182443283	5.160909016	2.125854212	6.03E-05	0.000286472
HGD	16.4810974	45.71895232	1.471980035	1.14E-07	1.48E-06

FIBCD1	1.69561616	3.775132036	1.154717483	1.92E-08	3.24E-07
PLA2G3	4.112792902	1.742820219	-1.238694672	1.57E-11	8.04E-10
LINC01781	2.22205488	0.959412842	-1.211670793	1.45E-08	2.57E-07
RHEX	3.966694423	1.967872678	-1.011300382	5.75E-06	3.96E-05
KLK6	3.53937199	13.42429249	1.923280752	1.56E-12	1.17E-10
PICSAR	0.800149049	3.342901434	2.062760146	0.000169028	0.000687829
C1orf189	4.803551141	1.788060929	-1.425705449	0.001237061	0.003684504
ARL14	3.621315716	15.07608101	2.057675587	3.18E-13	3.09E-11
CFAP73	4.853418251	1.79045929	-1.438671476	0.001726168	0.004882041
GTF2IP7	0.632813181	1.582301503	1.322172971	3.29E-16	8.74E-14
FAM166B	8.488642332	3.313025	-1.357384739	1.75E-05	0.000100318
FSCN1	69.83199975	154.6776608	1.147304659	1.71E-13	1.86E-11
SERPINB7	1.226501711	3.120352254	1.347159656	0.000753043	0.002431433
CFAP100	2.151507224	0.736342008	-1.54689977	6.05E-07	5.97E-06
DMBT1	105.2820098	41.72107582	-1.33541067	8.14E-06	5.30E-05
RHOXF1	1.386198796	0.630432377	-1.136720636	0.000712639	0.002323582
DTHD1	2.752111407	1.187935451	-1.212082426	0.000596706	0.002005442
MORN5	7.605587706	2.696837432	-1.495791177	0.004122285	0.010170465
SLC10A2	3.65373251	0.843200068	-2.115424129	0.002408706	0.006465929
AL121956.7	3.302399113	1.429791872	-1.207709333	0.00358385	0.009060957
RERGL	6.060390621	2.565781148	-1.240012667	8.95E-05	0.000401455
LAMC2	86.94806128	239.4472214	1.46148193	1.17E-16	3.68E-14
RXRG	2.393203612	1.104984904	-1.114916485	5.29E-07	5.33E-06
AC011379.2	1.652157161	0.819538456	-1.011467376	4.91E-07	5.01E-06
GAS2L2	3.914144677	1.713973361	-1.191352396	5.91E-05	0.000281698
MAGEA10	3.180894043	6.43848026	1.017287879	0.021222402	0.040341029
BLK	2.788663118	1.264034973	-1.141537281	7.42E-08	1.02E-06
CASC8	0.859892649	1.849866598	1.105192769	1.17E-08	2.13E-07
AC025154.2	8.73745019	3.527453142	-1.308585408	2.85E-10	9.07E-09
HPD	0.903703549	2.349710451	1.378561495	6.29E-05	0.000297234
NOS3	7.025848162	17.4412748	1.311761188	2.28E-07	2.63E-06
SPINK5	30.40282148	12.65984228	-1.263945785	0.002594937	0.006895062
SFRP5	3.357517997	1.655070014	-1.020502885	0.000185547	0.000744864
NECAB2	0.609732319	2.241276639	1.878072806	1.67E-12	1.23E-10
AC007686.1	0.622805577	1.948960724	1.64585125	9.31E-05	0.000415822

OMG	4.609323574	2.253872951	-1.032148854	0.000385378	0.001378985
CHST9	2.619468885	1.08967056	-1.265382294	0.012624982	0.026021969
AC110741.1	5.545232953	1.254048497	-2.144654927	0.000138421	0.000581943
FLACC1	1.375949113	0.636420765	-1.112374302	1.02E-05	6.41E-05
AL590004.3	0.426643156	1.698725615	1.993351031	2.30E-11	1.11E-09
TREML3P	1.577286565	4.113712363	1.382996126	1.74E-06	1.44E-05
ATP11AUN	1.400613054	0.683561407	-1.034915587	0.001754365	0.004945578
GPR1	0.613325665	1.447501981	1.238840094	6.57E-05	0.000308186
AC131206.1	1.88991635	4.059846243	1.10310271	0.000176429	0.000713571
AC002401.4	2.790938023	6.528416462	1.225983005	1.97E-05	0.00011112
ECRG4	6.468225222	2.84466571	-1.185110789	0.001132328	0.003418176
IRX6	8.115052345	3.049908538	-1.411834421	1.67E-06	1.39E-05
TNFRSF13B	1.929530101	0.878702186	-1.134803363	2.00E-08	3.36E-07
SLC1A7	28.41412218	13.42858224	-1.081301149	4.71E-09	9.74E-08
TAC4	7.882085551	1.559380669	-2.337604253	1.93E-12	1.39E-10
DYNLRB2	4.324862421	1.82252985	-1.246711796	0.002193388	0.005970582
GCKR	0.760391888	1.618738456	1.090054857	0.00010253	0.000449941
AL161431.1	0.907298923	4.939643238	2.444756997	2.53E-09	5.73E-08
AC126323.1	1.997196705	0.699806489	-1.512948484	5.93E-06	4.06E-05
CDHR3	6.948276426	3.142411544	-1.144783016	1.11E-05	6.88E-05
FGF12	1.099951711	2.641938388	1.264156633	0.000939232	0.002913239
LINC02313	0.996970976	2.409743579	1.273256227	0.000192262	0.000767715
AC112721.2	0.750357541	1.724057923	1.200158145	0.001814355	0.005091405
PKP2	6.424416603	13.6722627	1.089614668	1.93E-07	2.28E-06
RHOV	33.68785323	99.93777889	1.568801659	7.43E-10	2.05E-08
REG4	28.2238358	89.3276097	1.662192085	0.001393274	0.004076136
MYO16-AS1	1.52859493	3.556499044	1.218251625	0.004094616	0.010113766
GAP43	0.997860583	2.018701708	1.01651758	5.31E-07	5.35E-06
NCKAP5-AS2	1.412484791	0.701354372	-1.010019853	2.93E-07	3.25E-06
S100A7	14.02138783	35.65093484	1.346310753	6.66E-06	4.51E-05
C6orf118	3.162010203	1.04758306	-1.593777387	2.59E-05	0.000139614
C11orf97	3.023030292	1.009169467	-1.582826972	0.001917366	0.00532677
GCLC	20.50836274	61.1598849	1.576373371	1.04E-15	2.47E-13
LAMB3	201.6836323	407.4931796	1.014681907	2.94E-20	2.67E-17
CD19	7.297956971	3.601254645	-1.018993029	3.56E-09	7.59E-08

AC079949.2	0.936081559	2.698030055	1.52720028	2.16E-06	1.73E-05
NAMPTP1	7.25478365	18.79677712	1.373480823	4.66E-13	4.28E-11
ABCC2	1.455579848	19.47690335	3.742098432	2.46E-09	5.63E-08
SERPIND1	33.69508878	12.52690663	-1.427508125	0.002274842	0.006153498
